# Cell competition between anaplastic thyroid cancer and normal thyroid follicular cells exerts reciprocal stress response defining tumor suppressive effects of normal epithelial tissue

**DOI:** 10.1371/journal.pone.0249059

**Published:** 2021-04-01

**Authors:** Aidana Amrenova, Keiji Suzuki, Vladimir Saenko, Shunichi Yamashita, Norisato Mitsutake

**Affiliations:** 1 Life Sciences and Radiation Research, Graduate School of Biomedical Sciences Nagasaki University, Nagasaki, Japan; 2 Department of Radiation Medical Sciences, Nagasaki University Atomic Bomb Disease Institute, Nagasaki, Japan; 3 Fukushima Medical University, Fukushima, Japan; 4 Center for Advanced Radiation Emergency Medicine at the National Institutes for Quantum and Radiological Science and Technology, Chiba, Japan; Duke University School of Medicine, UNITED STATES

## Abstract

The microenvironment of an early-stage tumor, in which a small number of cancer cells is surrounded by a normal counterpart milieu, plays a crucial role in determining the fate of initiated cells. Here, we examined cell competition between anaplastic thyroid cancer cells and normal thyroid follicular cells using co-culture method. Cancer cells were grown until they formed small clusters, to which normal cells were added to create high-density co-culture condition. We found that co-culture with normal cells significantly suppressed the growth of cancer cell clusters through the activation of Akt-Skp2 pathway. In turn, cancer cells triggered apoptosis in the neighboring normal cells through local activation of ERK1/2. A bi-directional cell competition provides a suppressive mechanism of anaplastic thyroid cancer progression. Since the competitive effect was negated by terminal growth arrest caused by radiation exposure to normal cells, modulation of reciprocal stress response *in vivo* could be an intrinsic mechanism associated with tumor initiation, propagation, and metastasis.

## Introduction

At earlier stages of carcinogenesis, malignant transformation starts from a single cell that grows within an epithelial monolayer. The initiated cell continues to proliferate and accumulates genetic alterations, which give rise to malignant cancer cells. However, tissue microenvironment, in which multiple types of cells coexist, affects malignant propagation of initiated cells [1–7]. In fact, in a tissue microenvironment, the initiated cells are likely to interact with normal counterparts in a dynamic fashion over time. Such interactions may lead to a balance to maintain homeostasis at the cellular level through suppression of aberrant cell expansion by promoting cell death or to cell propagation if the balance is impaired [8]. Therefore, gaining a greater understanding of the mechanisms how the initiated and normal cells interact at a cellular level should provide deeper insights into the complexity of cancer development in vivo.

Cell competition was originally identified more than 40 years ago in developing Drosophila melanogaster tissues, where it was considered to play a role of a quality control system in the growing embryo by eradicating undesirable cells during development [9]. Since then cell competition has been confirmed in a variety of physiological processes, including embryogenesis, morphogenesis, and aging [10–15]. In particular, it is now believed that cell competition is a general feature of tissues and organs for eliminating the variants in specified cell lineages, which is critical for tissue homeostasis [15–19]. In other words, it works as a natural selection process between normal and mutated cells [10–13]. In most cases, cells showing growth-advantage win out over their neighbours with growth-disadvantage in host tissue. Accordingly, the less-fit cells or namely, ‘losers’ are eliminated by apoptosis, while more fit cells or ‘winners’ proliferate and dominate neighborhood [12, 13, 15].

Thus, cell competition is now recognized as a critical player in the development of diseases, such as cancer [8, 20–25]. In carcinogenesis, Myc-mediated cell competition is the most well-studied example [26–30]. Since cells showing higher expression of Myc were demonstrated to cause competitive cell elimination in *Drosophila* [27, 28], many types of cancer cells, which overexpress c-Myc protein [31–35], have been though to enable their expansion in tissues with the aid of c-Myc-mediated super-competition [36, 37]. p53, which is mutated in more than 50% of cancers [38–41], has also been shown to play a role in cell competition, and cells with mutated p53 function outcompeted the wild-type p53 cells [42, 43].

Thyroid cancer is the most frequent endocrine malignancy [44–47]. Amongst three major types of thyroid cancers, papillary, follicular, and anaplastic thyroid cancers, anaplastic thyroid cancers (ATC) is a rare but highly aggressive disease [45, 48–50]. Treatment modalities such as surgical resection, chemotherapy and radiation therapies have not been effective [45, 47, 50–52]. Recently, the molecular abnormalities in ATC have been studied on RNAs, genome and protein levels, which uncovered the most prevalent mutations were those of p53, BRAF and RAS, suggesting that increased cell competitiveness could be involved in the malignant phenotype [53–55]. Considering that cancer cell growth in thyroid follicles is affected by the interaction with the environment and that cell competition could be one of such actual mechanisms, we set out to evaluate its role in a model employing ATC cells (ACT1 cell line) and normal thyroid follicular epithelial cells (NTECs). Since cell competition is a dynamic process, live-cell imaging of co-culture, which mimicked the small clusters of cancer cells surrounded by normal cells, was used. The results demonstrated suppressive growth of ACT1 cells in competitive interaction with NTECs, and at the same time, NTECs were outcompeted by ACT1 cells and underwent elimination through apoptosis. Regional elimination of NTECs required direct contact with ACT1 cells and local ERK activation. Thus, our results demonstrated that cell competition is a bi-directional phenomenon and that it provides a suppressive mechanism of anaplastic thyroid cancer progression. Since cell competition was negated by terminal growth arrest of normal cells, alteration of reciprocal stress response *in vivo* could be an intrinsic mechanism associated with tumor initiation, propagation, and metastasis.

## Materials and methods

### Cell culture

Normal human thyroid follicular epithelial cells (NTECs) were spontaneously immortalized cells derived from human thyroid gland [56], and anaplastic thyroid carcinoma cell lines (ACT1) were obtained from Dr. N. Onoda (Osaka City University, Osaka, Japan; originally established by Dr. S. Ohata of Tokushima University [57]. Cells were cultured in DMEM (Wako, Tokyo) supplemented with 10% fetal bovine serum (FBS) (TRACE Bio) under standard conditions in a humidified incubator at 37°С with 5% CO_2_.

NTECs and ACT1 cells were checked their human origin by chromosome analysis, and mycoplasma contamination was routinely inspected by DAPI staining.

### Irradiation

NTECs cultured in a culture flask were exposed to 10 Gy of γ-rays from a γ-ray irradiator equipped with a ^137^Cs source (Pony Industry Co., Ltd, Osaka) at a dose rate of 1 Gy/min.

### Cell labelling

ACT1 was labelled with the Qtracker 655 cell labeling reagent (ThermoFisher Scientific, Tokyo, Japan). Cells were cultured onto sterilized 22 x 22 mm glass slips (Matsunami, Tokyo, Japan) placed in the 35-mm culture dishes. For labelling, a 10 nM solution was prepared according to the manufacturer’s protocol and added to the dishes with ACT1 cells for 1 hr at 37°С followed by two washes with fresh growth medium.

NTECs were transfected with the pHOS-H2B-GFP plasmid (BD Bioscience, Tokyo) from which the green fluorescent protein (GFP)-tagged histone H2B is produced. Introduction of the plasmid into NTEC was performed by electroporation (Electric Cell Fuser, ECF2001, Wakenyaku, Tokyo). Exponentially growing cells were collected by trypsinization, suspended in PBS, and 200 μl of cell suspension was added to a electroporation cuvette (0.2 cm) with plasmid DNA (1 μg). Three pulses with an intensity of 400V/cm with a constant pulse duration of 1 msec were used. Immediate after the pulse, cell suspension was transfer to 100-mm dishes with 10 ml of DMEM medium and cultured for three days. Cells were then collected by trypsinization, replated onto three 100-mm dishes with 10 ml of DMEM medium containing 400 μg/ml G418 (WAKO Pure Chemicals, Osaka), and cultured for further 7 day before G418-resistant colonies were formed. Colonies were randomly isolated, and independent clones were cultured in T25 flasks. A part of each clone was replated onto glass-bottomed dishes (AGC Techno Glass Co., Ltd, Tokyo), and GFP expression was checked under a fluorescent microscope (BZ-9000, KEYENCE, Osaka) to select GFP-H2B-positive clones. The clone, which expressed the strongest GFP fluorescence, was named GFP-NTECs.

### Co-culture of ACT1 and NTEC cells

Exponentially growing ACT1 cells were plated on glass coverslips at low cell density (400 cells/slip) and incubated for about 5 days until they formed small cell clusters. Then, NTECs were added to the culture (10^5^ cells/slip), and incubated for further 3–5 days.

### Time-lapse microscopy

Time-lapse imaging was performed by BioStation-ID (GE Helthcare Bioscience, Tokyo, Japan) with a x10 objective lens. Images were captured in every 5 min for up to 72 h. In each experiment, at least ten fields were imaged in GFP fluorescence channel along with phase contrast.

### Live-cell imaging of apoptosis

For the detection of apoptosis in living cells, CellEvent Caspase-3/7 Green Detection Reagent (ThermoFisher Scientific, Tokyo, Japan) was used. The reagent (2 μl) was diluted in growth medium and added to the dishes containing ATC1 clusters and NTECs. The cultures were incubated for 30 minutes according to the manufacturer’s instruction, and apoptotic cells with activated caspase-3/7, which showed bright green fluorescence, were analyzed by time-lapse microscopy using Biostation-ID.

### Immunofluorescence

For immunofluorescence, the cells on glass coverslips were fixed in 4% formaldehyde for 10 minutes on ice, followed by the permeabilization with 0.05% Triton X-100 for 5 min on ice. After extensive wash with PBS, primary antibodies diluted in TBS-DT (20 mM Tris-HCl, pH7.6, 137 mM NaCl, 0.1% Tween 20, 125 μg/ml ampicillin, 5% skim milk) were treated for 2 hr at 37°C, followed by the Alexa Fluor-labeled secondary antibodies for 1 hr at 37 ˚C. After extensive washes with PBS, coverslips were mounted on glass slides with 10% glycerol/PBS containing 1 μg/ml DAPI. The primary antibodies used in the experiments were rabbit anti-phospho ERK1/2 antibody (1:500 dilution, #4370, Cell Signaling Technology Japan, Tokyo), rabbit anti-c-myc antibody (1:500 dilution, ab32071, Abcam Japan, Tokyo), mouse anti-CDH1 antibody (1:500 dilution, 610182, BD Biosciences, San Jose, CA), mouse anti-phospho H2AX antibody (1:500 dilution, 613402, BioLegend, San Diego, USA), rabbit anti-Ki-67 antibody (1:1000 dilution, ab16667, Abcam Japan, Tokyo), chicken anti-Vimentin antibody (1:2000 dilution, ab92547, Abcam Japan, Tokyo), rabbit anti-active YAP1 antibody (1:200 dilution, ab205270, Abcam Japan, Tokyo), and rabbit anti-53BP1 antibody (1:500 dilution, A300-272A, Bethyl Laboratories Inc., Montgomery, TX). The secondary antibodies used were goat Alexa Fluor 488-labeled anti-mouse IgG (1:1000 dilution, A11001, Thermo Fisher Scientific), goat Alexa Fluor 555-labed anti-rabbit IgG (1:1000 dilution, A21428, Thermo Fisher Scientific, Tokyo), and goat Alexa Fluor 647-labed anti-chicken IgY (1:1000 dilution, A150171, Thermo Fisher Scientific, Tokyo). Images were captured by a fluorescence microscope (DM6000B, Leica Japan) and analyzed by image processing system (FW4000, Leica Japan).

### Image analysis

Images were taken under a fluorescent microscope (BZ-9000) and the fluorescence area corresponding to a cell cluster size was determined using Image J software [58]. Briefly, the selection tool was used to set the area of interest, and the image was converted to Grayscale. The pixels were highlighted using the threshold dialog, and the total number of pixels was defined by selecting the menu command "measurements". For analyzing phosphorylation of ERK1/2, the distance from ACT1 cell cluster was marked with the straight selection tool. Next, the colour images were splitted using the menu command "Image-Type-Colour-Split channels", and using the blue channel, which is for the DAPI staining, the total number of NTEC cells per distance was calculated. The number of NTEC cells with phosphorylated ERK1/2 was quantified with the merged image between the blue and red channels using the menu command "Image-Colour-Merge channels", and cells positive for both channels were counted.

### Western blotting

Cells were treated with lysed in RIPA buffer (50 mM Tris-HCl (pH 7.2), 150 mM NaCl, 1% NP-40, 1% sodium deoxycholate, and 0.1% SDS). The cell lysate was cleared by centrifugation at 15,000 rpm for 10 min at 4°C, and the supernatant was used as the total cellular protein. Protein concentration was determined by the BCA protein assay (Pierce, Rockford, IL). Protein samples (8 or 16 μg) were electrophoresed on a SDS-polyacrylamide gel (ATTO Corporation, Tokyo) and were transferred to a polyvinyl difluoride (PVDF) membrane (Millipore Japan, Tokyo) in a transfer buffer (100 mM Tris, 192 mM glycine). After incubation with blocking solution (10% skim milk in TBS-T buffer (20 mM Tric-HCl, pH7.6, 137 mM NaCl containing 0.1% Tween-20)) for one hour, the membrane was incubated with the primary antibodies for 2 hours followed by the secondary goat anti-mouse IgG or goat anti-rabbit IgG antibodies conjugated with alkaline phosphatase (BioRad Laboratories, Inc, Tokyo, Japan). The bands were visualized with nitroblue tetrazolium/5-bromo-4-chloro-3-indolyl phosphate. The primary antibodies used in this study were rabbit anti-ERK1/2 (1:500 dilution, #4695, Cell Signaling Technology Japan, Tokyo), rabbit anti-phosphorylated ERK1/2 (1:500 dilution, #4370, Cell Signaling Technology Japan, Tokyo), rabbit anti-p38 (1:500 dilution, #9212, Cell Signaling Technology Japan, Tokyo), rabbit anti-phosphorylated p38 (1:500 dilution, #4511, Cell Signaling Technology Japan, Tokyo), rabbit anti-phosphorylated RB at S780 (1:500 dilution, #8180, Cell Signaling Technology Japan, Tokyo), rabbit anti-RB (1:500 dilution, #9313, Cell Signaling Technology Japan, Tokyo), mouse anti-p21^WAF1/Cip1^ (1:500 dilution, ab107099, Abcam plc, Cambridge, UK), rabbit anti-p27^Kip1^ (1:500 dilution, ab32034, Abcam plc, Cambridge, UK), mouse anti-p16^INK4a^ (1:500 dilution, ab117443, Abcam plc, Cambridge, UK), rabbit anti-phosphorylated Skp2 at S64 (1:500 dilution, #14865, Cell Signaling Technology Japan, Tokyo), rabbit anti-Akt (1:500 dilution, #4691, Cell Signaling Technology Japan, Tokyo), rabbit anti-phosphorylated Akt at S473 and T308 (1:500 dilution, #2965 and #4060, Cell Signaling Technology Japan, Tokyo), rabbit anti-c-myc antibody (1:500 dilution, ab32071, Abcam Japan, Tokyo), mouse anti-p53 antibody (1:500, BP53-12, dilution, 628201, BioLegend, San Diego, USA), goat anti-Scrib (1:500 dilution, sc-11049, Santa Cruz Biotechnology Japan, Tokyo), and mouse anti-β-Actin (1:500 dilution, 2F1-1, BioLegend, San Diego, USA).

### Senescence associated β-galactosidase staining

Cells plated on cover glass were washed with PBS and treated with a fixative (2% formaldehyde, 0.2% glutaraldehyde in PBS) for 5 min at room temperature. After a wash with PBS, cells were incubated with staining solution (40 mM citric acid/sodium phosphate, pH 6.0, 5 mM Potassium ferricyanide, 5 mM Potassium ferrocyanide, 150 mM NaCl, 2 mM MgCl_2_ containing 1 mg/ml 5-bromo-4-chloro-3-indolyl β-D-galactoside, WAKO Pure Chemicals, Osaka) for 12–24 hours at 37°C. After extensive washes with PBS, coverslips were mounted on glass slides with 10% glycerol/PBS and stored at 4°C.

### Statistics

For each experiment, at least three independent repeats were carried out. The non-parametric Mann-Whitney test was used for colony size comparison. Weighted linear regression was used to analyze region-specific phosphorylation of ERK1/2. Statistical calculations were performed using JMP 15 Pro software. The *p*-value < 0.05 were considered indicative of statistical significance.

## Results

### Cell competition between anaplastic thyroid cancer cells and normal thyroid follicular cells

**Fig 1:**
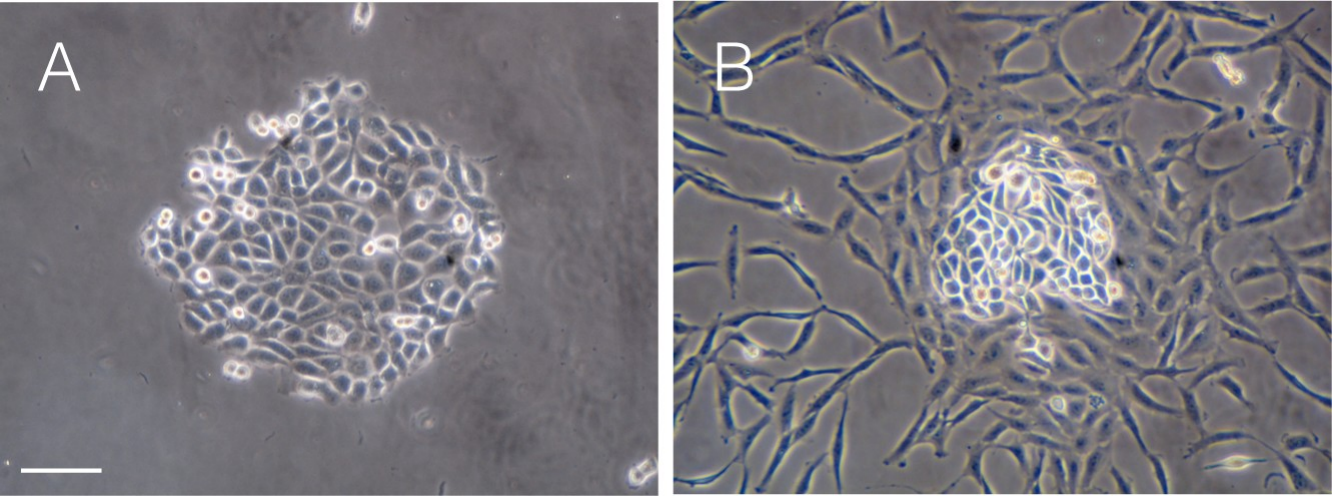
Co-culture of ACT1 cell cluster with NTECs. ACT1 cells were cultured for 5 days until they formed small cell clusters of approximately 500 μm in diameter. Then, NTECs were added to establish co-cultures. (A) ACT1 cluster in monoculture at Day 7. (B) ACT1 cluster with NTECs two days after co-culture. NTECs were gradually crowded around ACT1 cell clusters. The bar in (A) indicates 100 μm.

Cell competition was reproduced by establishing a co-culture system between anaplastic thyroid cancer cell line, ACT1, and normal thyroid follicular epithelial cells (NTECs). ACT1 cells plated at a low density grow clonally and form densely packed cell clusters by Day 5 (Fig 1A), and then, NTECs were added to the culture. We observed that all ACT1 clusters were surrounded by NTECs (Fig 1B).

**Fig 2:**
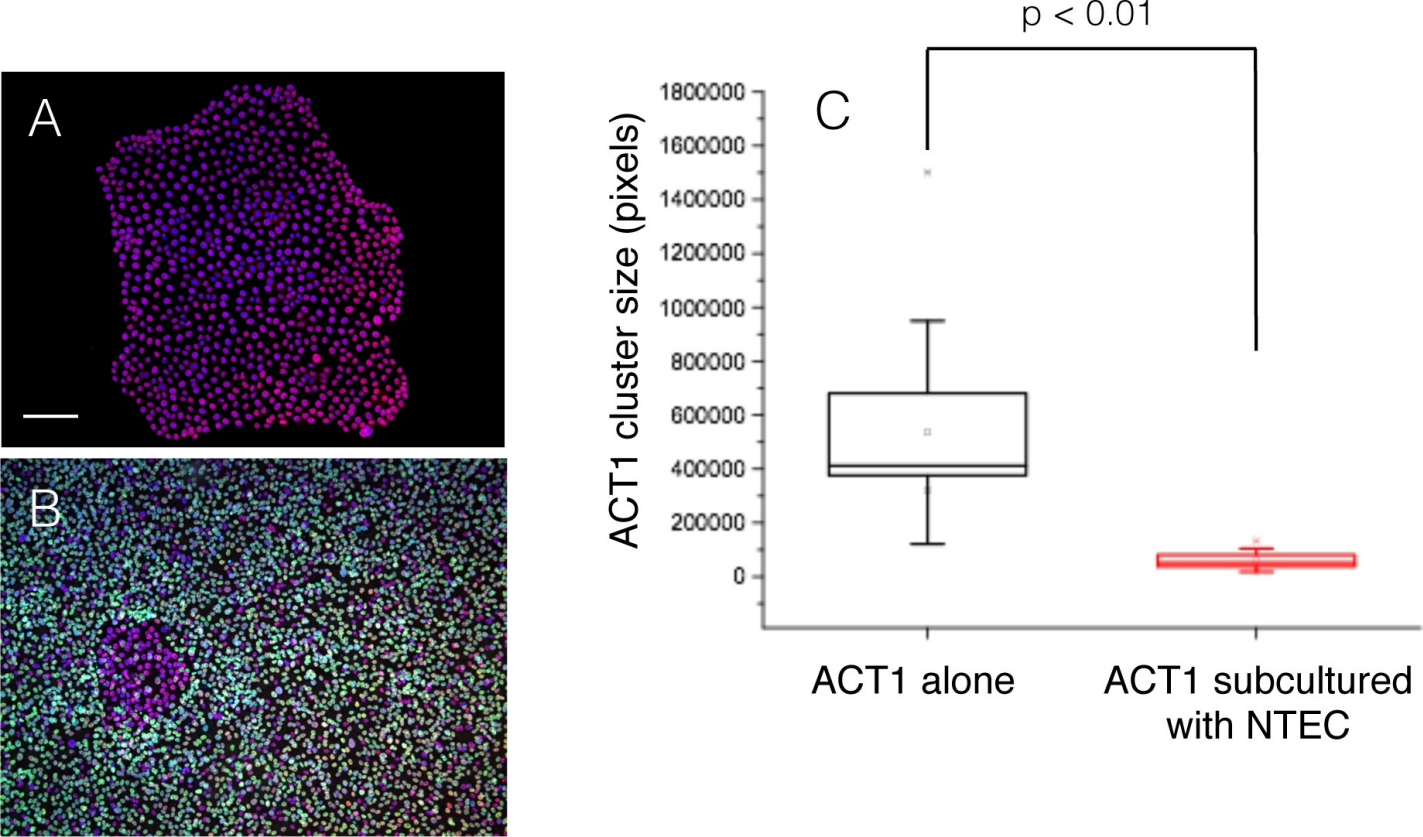
Suppression of ACT1 cell growth by competition with NTECs. ACT1 cells were cultured for 5 days until they formed small cell clusters, and then GFP-NTECs were added and cultured for 3 more days. ACT1 cluster sizes at Day 8 were measured in ATC1 monocultures (A) and in co-cultures (B), and their sizes were compared (C) as described in Materials and Methods. ACT1 clusters and NTECs were fixed and stained with anti-53BP1 antibody (red fluorescence). The bar indicates 100 μm.

The time-lapse analysis demonstrated that NTECs added to the culture are gradually accumulated around ACT1 clusters even at very low cell density, and crowding around ACT1 clusters becomes obvious approximately 6–8 hrs later (S1 Fig). After 3–5 days incubation, NTECs fully occupied the space between the ACT1 clusters (S2 Fig), and the growth of ACT1 clusters seemed to be suppressed, as we observed little change in the sizes of the clusters among NTECs compared with ATC1 clusters in monocluture. To confirm the suppressive effects GFP-tagged NTECs, which enable to discriminate normal cells in co-culture, were used (Fig 2).

ACT1 cluster sizes in monocultures (Fig 2A) are obviously larger than those in co-cultures (Fig 2B), and the difference is statistically significant (Fig 2C), demonstrating that cell competition between neighbouring NTECs and ACT1 clusters retarded ACT1 cluster growth. According to the results of immunofluorescence using anti-Ki-67 antibody, ACT1 clusters lose proliferating potential. Particularly, ACT1 cells in the center of clusters became negative for Ki-67, while all of ACT1 cells are positive for Ki-67 in monoculture (S3 Fig). This confirms that cell competition between NTECs and ACT1 clusters retarded cell cycle progression of ACT1 cells.

**Fig 3:**
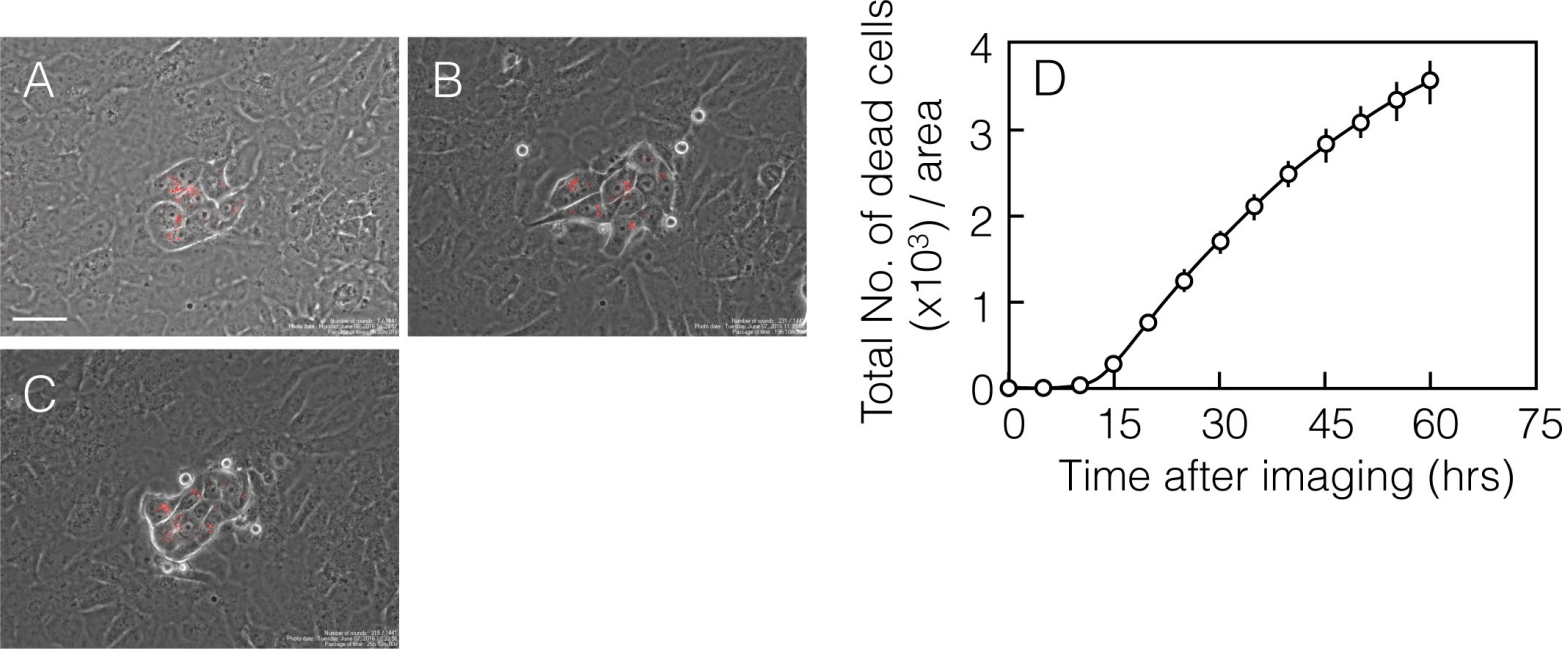
Time-lapse analysis demonstrates NTEC-specific cell death. ACT1 cells labeled with Qtracker 655 were incubated for 3 days before NTECs were added. Time-lapse imaging was started 48 hours after co-culture (A). Representative images of co-cultures at 18 h (B) and 24 h (C) thereafter. Spatiotemporal occurrence of dead NTECs, which appered as small round-shaped cells within 100 μm distance from the ACT1 clusters, was recorded and summed every 5 hours. Total number of dead cells indicates the summed number of dead cells obtained every 5 hours (D). The bar indicates 40 μm.

In contrast to the growth arrest of ACT1 clusters, the time-lapse analysis also exhibited regional death of NTECs in the areas close to ACT1 clusters (Fig 3 and S1 Movie). During the first approximately 48 hours after inoculating NTECs, growth of NTECs was more than that of ACT1, so that dividing cells were observed more in NTECs, and then, morphologically distinct cells, which were small round but not the mitotic cells, were emerging in proximity to ACT1 clusters (Fig 3B and 3C).

**Fig 4:**
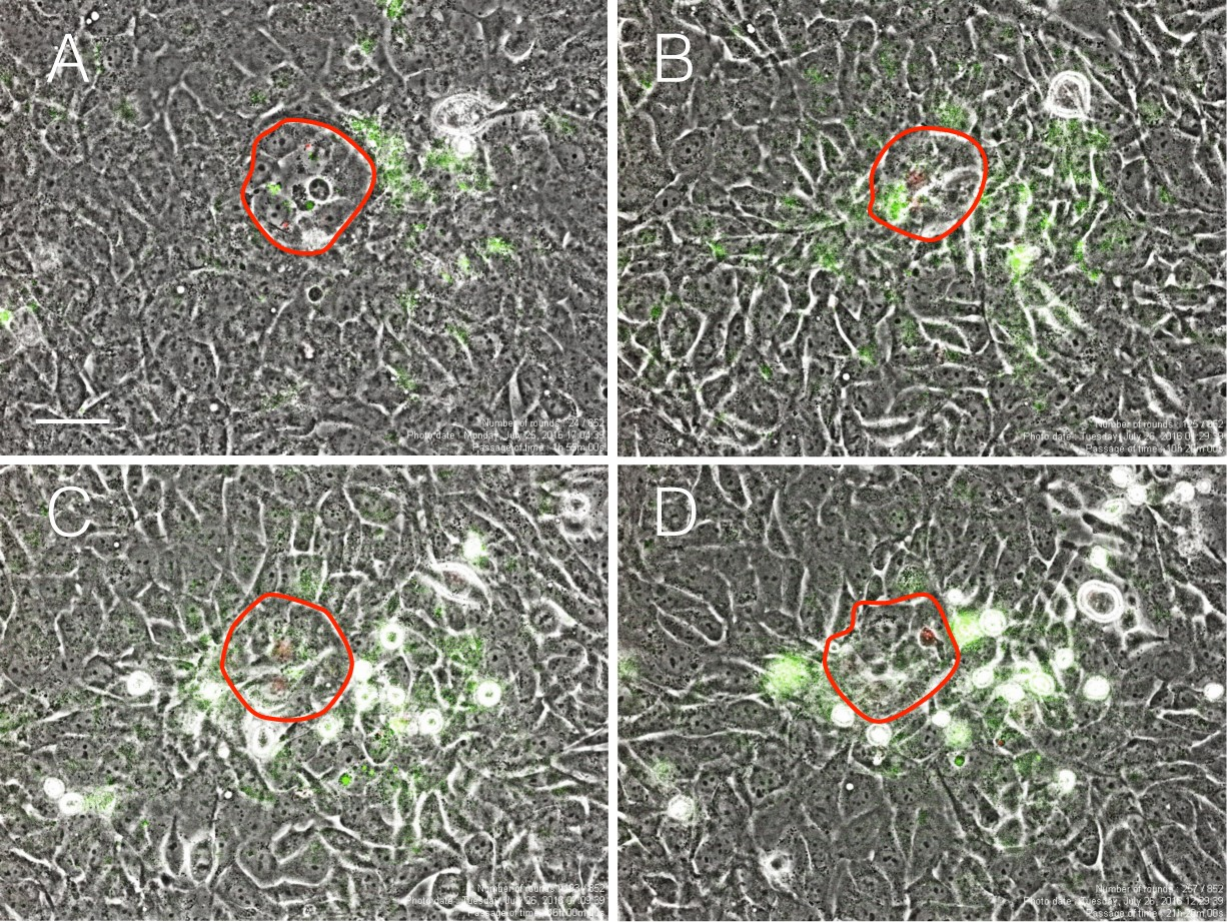
Time-lapse analysis demonstrates NTEC-specific apoptosis. ACT1 cells labeled with Qtracker 655 (red fluorescence) were incubated for 3 days before NTECs were added. Time-lapse imaging was started 48 hours after co-culture at which time point CellEvent Caspase-3/7 Green detection reagent was added and incubated for 30 min (A). The freeform shape indicated by red lined corresponds to ATC1 cluster borders. Representative images of cocultures with CellEvent Caspase-3/7 Green detection reagent incubated for further 6 hours (B), 12 hours (C) and 16 hours (D). The bar indicates 50 μm.

Detailed time-lapse analysis revealed that cell blebbing, which is one of the defined features of apoptosis, preceded the cells being round-shaped (S4 Fig). It was found that small round-shaped cells were subsequently ruptured. The time-lapse analysis also confirmed that they were derived from Qtracker-negative cells (S4 Fig). Small round-shaped cells were also GFP-tagged and positive for vimentin, which is a marker for NTECs (S5 Fig), confirming that cell death was induced in NTECs. Temporal analysis of time-lapse images shows that dead cells were appearing continuously over 60 hours (Fig 3D), while none of them was detectable during the first approximately 12 hours. Judging from their morphology, NTECs was likely dead by apoptosis, which was confirmed by live-imaging using cell-permeable fluorescent substrates for Caspase-3 and 7. As shown in Fig 4, NTECs in the areas close to ACT1 clusters are marked by green fluorescence (Fig 4B and 4C), and those cells become round-shaped cells afterwards (Fig 4C and 4D), indicating that Caspase-3/7-mediated apoptosis is involved in NTEC death (S2 Movie). Apoptotic cell death is also confirmed by an immunofluorescent analysis using γ-H2AX antibody, which depicts DNA fragmentation (S6 Fig).

**Fig 5:**
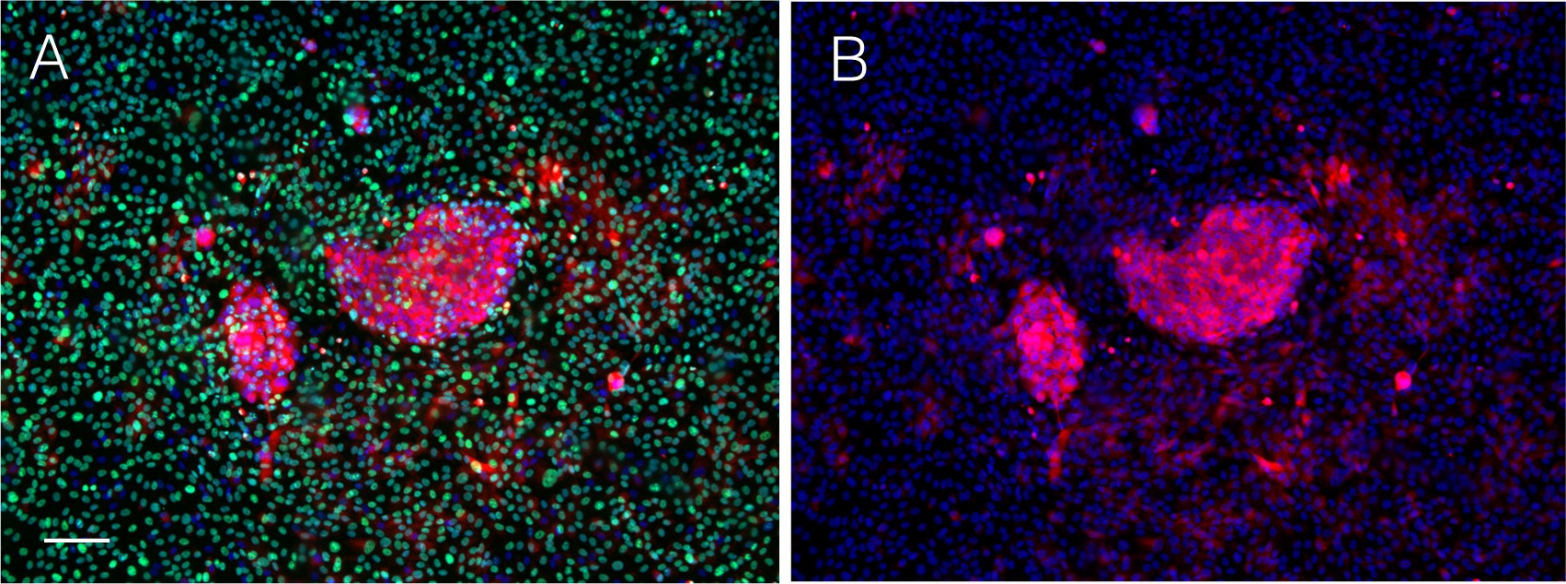
Detection of phosphorylation of ERK1/2. ACT1 cell clusters were co-cultured with GFP-NTECs for 5 days, fixed with formaldehyde, and stained with anti-phosphorylated ERK1/2 antibody (red) and anti-Ki-67 antibody (green). (A) Merged image. (B) Red fluorescence image showing regional phosphorylation of ERK1/2 in NTECs. The bar indicates 100 μm.

To uncover the mechanisms underlying position-specific cell death, we performed an immunofluorescent analysis using antibodies against phosphorylated forms of MAP kinases. Among ERK1/2, p38 and JNK1/2, only ERK1/2 was found to be phosphorylated in NTECs. whereas augmented phosphorylation is common in ACT1 clusters (Fig 5).

**Fig 6:**
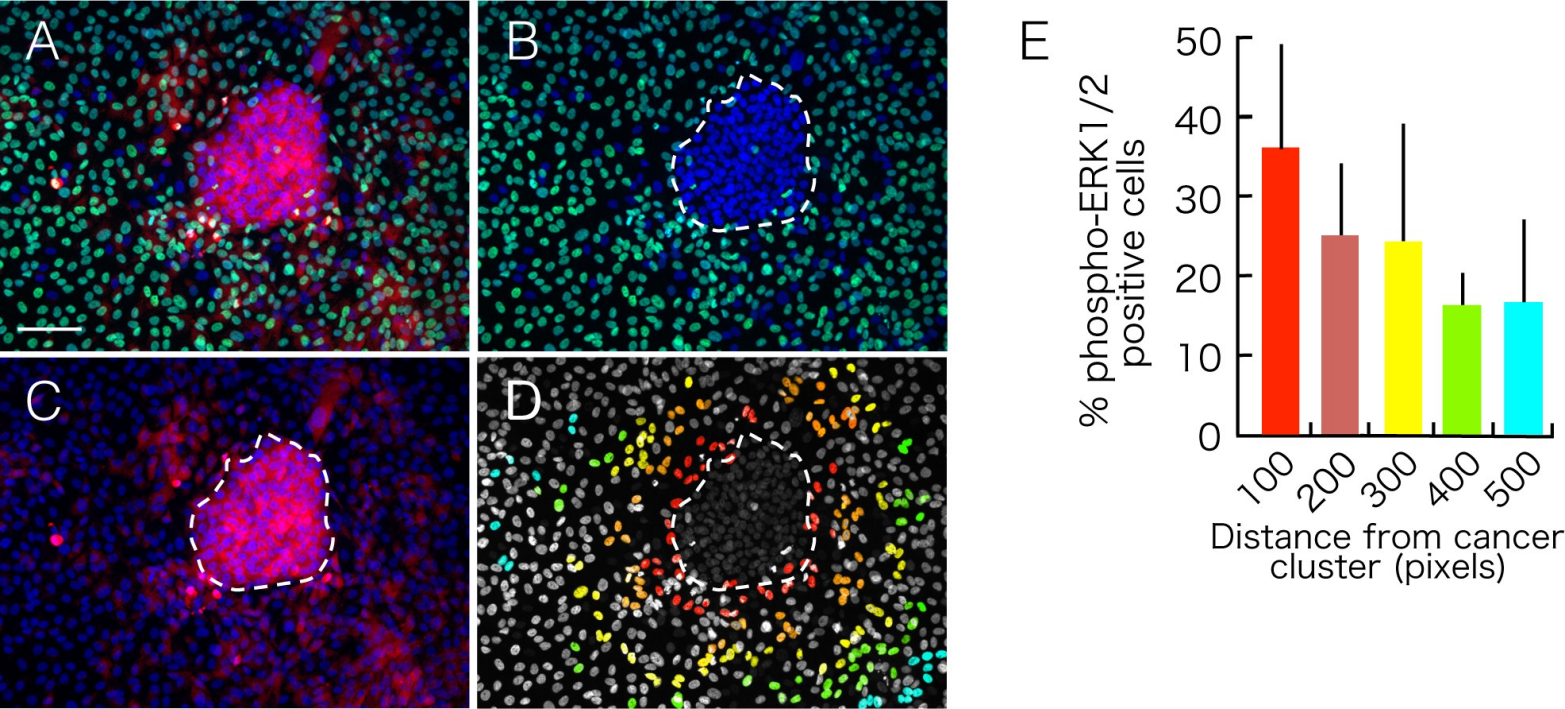
Analysis of region-specific phosphorylation of ERK1/2. ACT1 cell clusters were co-cultured with GFP-NTECs for 5 days, fixed with formaldehyde, and stained with anti-phosphorylated ERK1/2 antibody. (A) Merged image. (B) Green fluorescence image showing GFP-NTECs. Area designated with a white dashed line indicates ACT1 cell cluster. (C) Red fluorescence image showing phosphorylation of ERK1/2. (D) NTECs with phosphorylated ERK1/2 signals zoned according to their distance from the ACT1 cluster. Red cells are phosphorylated ERK1/2-positive within 100 pixels from the cluster, orange 100–200 pixels, yellow 200–300 pixels, green 300–400 pixels, cyan 400–500 pixels. Percentage of NTECs with phosphorylated ERK1/2 is indicated in (E). The bar in (A) indicates 100 μm.

Importantly, NTECs with phosphorylated ERK1/2 are localized to the areas close to ACT clusters, and notably, round-shaped dead NTECs are phosphorylated ERK1/2-positive (S7 Fig). As shown in Fig 5, phospho-ERK1/2 positivity was observed more in NTECs closer to the clusters, so that the frequency of phospho-ERK1/2-positive NTECs is determined in the areas every 100 pixels from the ACT1 cluster border (Figs 6 and S8). Analysis of distance-dependent ERK1/2 phosphorylation revealed the highest frequency is observed in NTECs closest to the ACT1 clusters (Fig 6E), however, even NTECs within the area more than 500 pixels apart from the ACT1 cluster border, which are equivalent to more than 300 μm apart from the clusters, still show phosphorylation of ERK1/2.

### Molecular pathways involved in cell competition

Since the growth of ACT1 clusters was significantly retarded in co-cultures with NTECs, we further analysed molecular changes in ACT1 cells as well as NTECs using discriminatory cell collection technique (S9 Fig). As the substrate attachment of ACT1 cells is much stronger than that of NTECs, we briefly trypsinized co-cultures without PBS wash and collected NTECs. Then, cells were trypsinized again to collect cancer cells remaining in the dish. Purity of the NTEC and ACT1 cell population was checked by using the GFP positivity determined under the fluorescence microscope, and more than 99.9% of the first collected cells and less than 0.5% of the second collected cells were positive for GFP, respectively.

**Fig 7:**
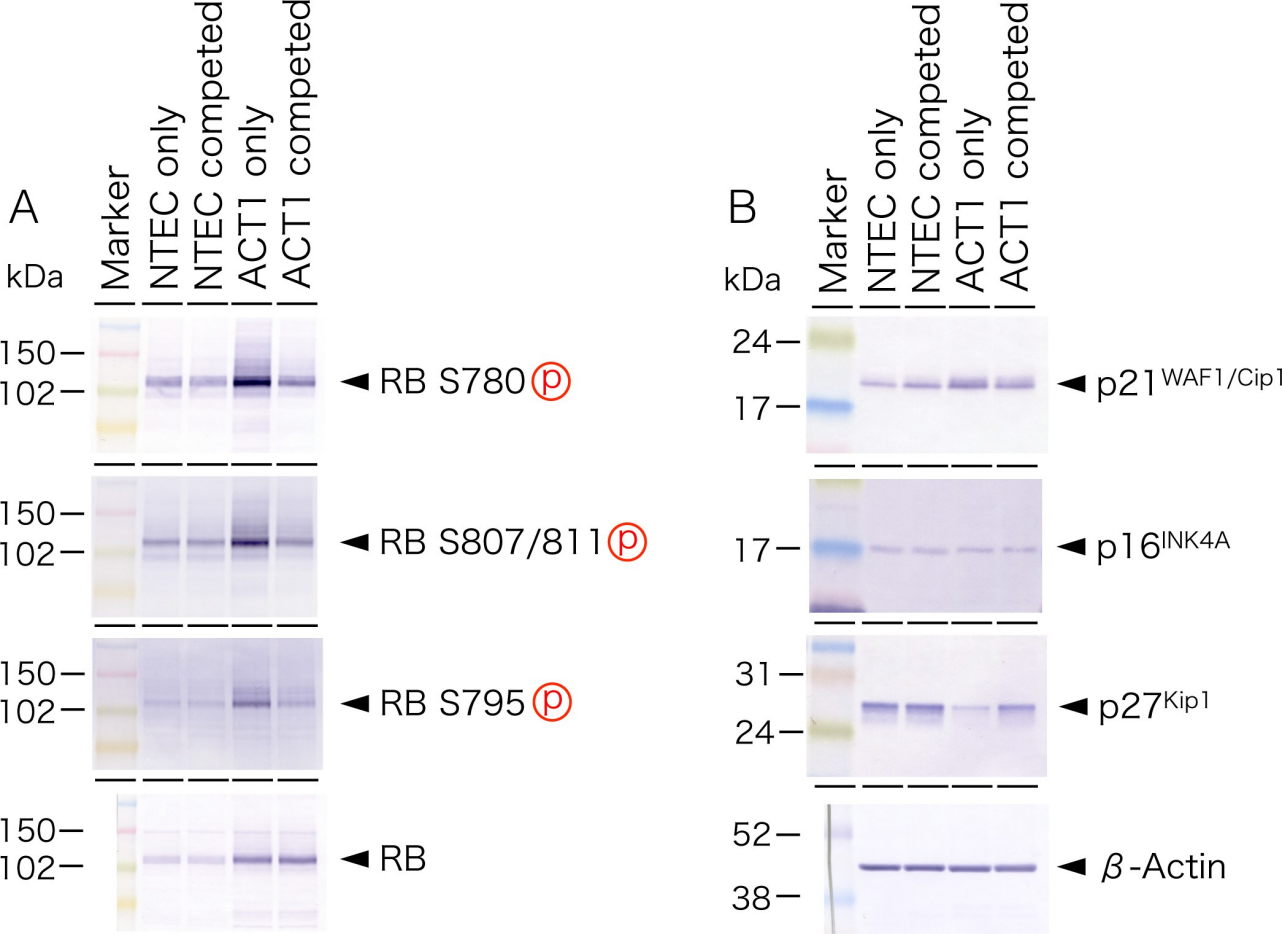
Western blot analysis of RB and CKIs. Total protein was extracted from both NTECs and ACT1 cells cultured either alone (NTEC or ACT1 only, respectively) or from cell co-cultures (NTEC or ACT1 competed, respectively) and subjected to western blot analysis with indicated antibodies.

Western blot analysis demonstrated that multiple phosphorylations of RB were significantly reduced in ACT1 cells that competed with NTECs, while total RB protein level was not changed (Fig 7A). As RB phosphorylations are targeted by several Cyclin/Cdk kinases, we examined the levels of their inhibitors. Among p21^WAF1/Cip1^, p16^INK4A^, and p27^Kip1^, the p27^Kip1^ protein, which are expressed in ACT1 cells at lower level as compared with NTECs, was profoundly upregulated in competed ACT1 cells (Fig 7B).

**Fig 8:**
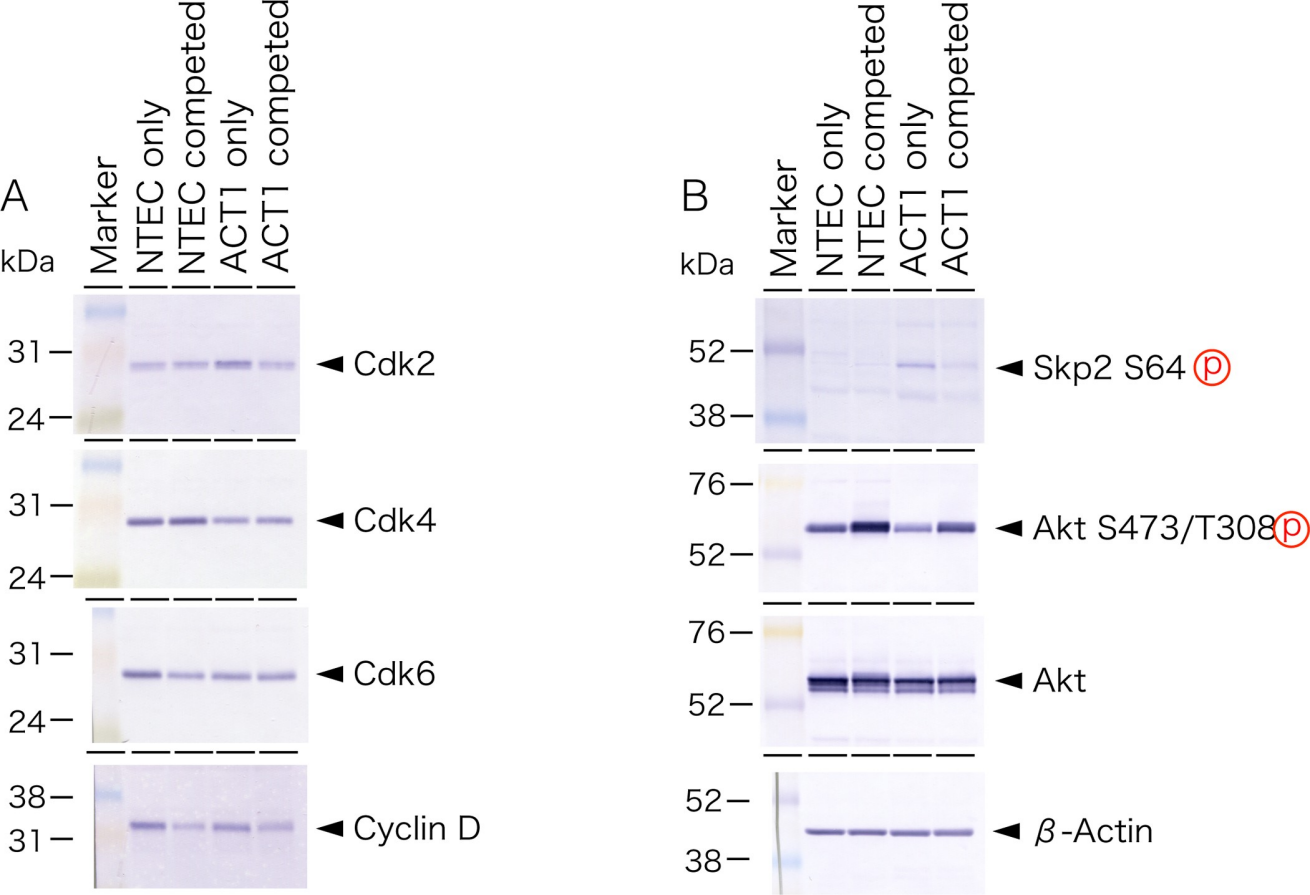
Western blot analysis of cell cycle regulators and Akt. Total protein was extracted from both NTECs and ACT1 cells cultured either alone (NTEC or ACT1 only, respectively) or from cell co-cultures (NTEC or ACT1 competed, respectively) and subjected to western blot analysis with indicated antibodies.

We also observed the decreased expression of Cdk2 and Cyclin D in competed ACT1 cells (Fig 8A), indicating that upregulation of p27^Kip1^ and downregulation of Cyclin D/Cdk2 could be involved in reduced RB phosphorylation. Competed ACT1 cells also displayed lower level of phosphorylated Skp2 at serine 64 and increased level of phosphorylated forms of Akt (Fig 8B).

**Fig 9:**
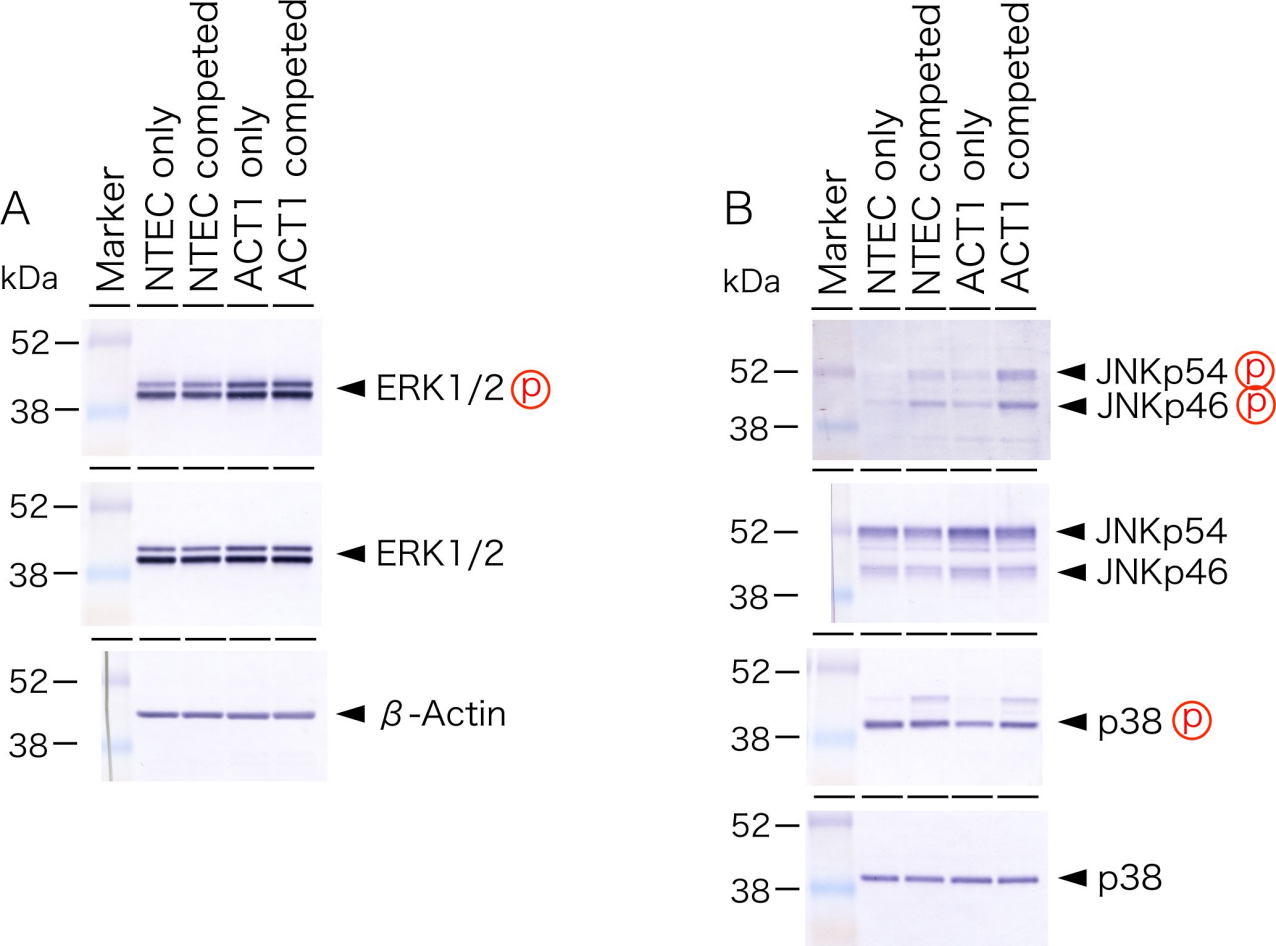
Western blot analysis of MAP-kinase pathway. Total protein was extracted from both NTECs and ACT1 cells cultured either alone (NTEC or ACT1 only, respectively) or from cell co-cultures (NTEC or ACT1 competed, respectively) and subjected to western blot analysis with indicated antibodies.

In agreement with the immunofluorescence results, phosphorylation of ERK1/2 was higher in ACT1 cells compared with NTECs, however, the level is not changed by cell competition (Fig 9A). As we observed an increased ERK1/2 phosphorylation in NTECs which were close to ACT1 clusters, we examined phosphorylation of ERK1/2 in NTECs from bulk co-culture. Despite regional phosphorylation was detected on immunofluorescence analysis, we did not detect changes in ERK1/2 phosphorylation under such conditions (Fig 9A). We also observed increased phosphorylation of JNK1/2 in both types of competed cells (Fig 9B).

### Effect of terminal growth arrest of NTECs on ACT1 cell growth

To determine whether the proliferation of NTECs is required for cell competition, NTECs were exposed to 10 Gy of γ-rays, which induces senescence-like terminal growth arrest as judged by the expression of senescence associated β-galactosidase activity (S10 Fig).

**Fig 10:**
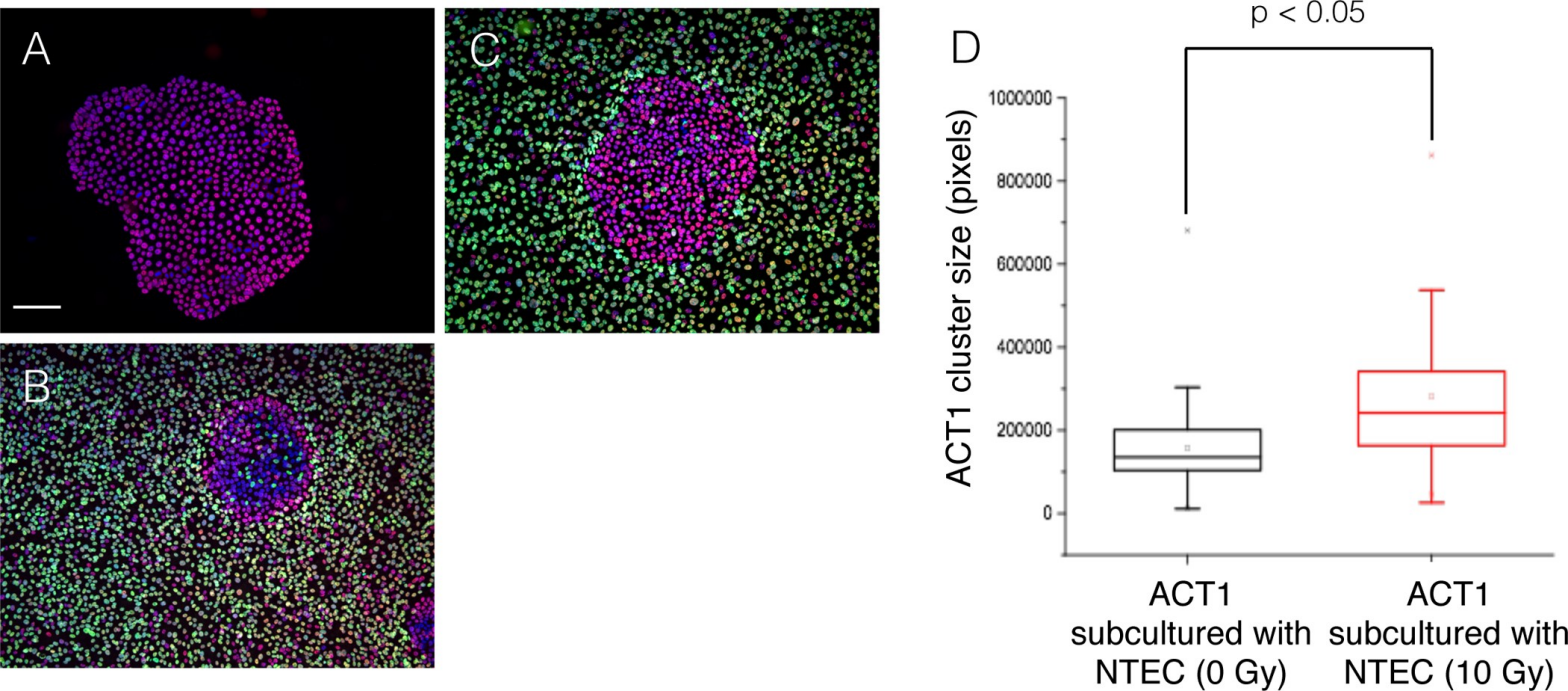
Reduced cell competition between terminally arrested NTECs and ACT1 cell cluster. ACT1 cells were cultured for 5 days until they formed small cell clusters and then GFP-NTECs irradiated with 10 Gy of γ-rays were added and cultured for 3 more days. ACT1 cluster sizes were measured 3 days later (on Day 8). ACT1 clusters and GFP-NTECs were fixed and stained with anti-53BP1 antibody (red fluorescence). (A) ACT1 cluster in monoculture. (B) ACT1 cluster co-cultured with GFP-NTECs at Day 8. (C) ACT1 cluster with 10 Gy-irradiated GFP-NTECs at Day 8. (D) Comparison of the ACT1 cluster sizes. Statistical difference was evaluated by Mann-Whitney test. The bar indicates 100 μm.

NTECs were exposed to γ-rays and incubated for 3 days before adding to ACT1 clusters. The quantity equivalent to that inducing competition (10^6^ cells) were added to ACT1 cluster, and ACT1 cluster sizes were measured 3 days later. It is apparent that the cluster sizes are appeared to be significantly greater than those observed in co-cultures with unexposed NTECs (Fig 10). Immunofluoresent analysis reveals that Ki-67 positivity, which was significantly reduced in competed ACT1 clusters, is remarkably recovered under this condition (S11 Fig).

## Discussion

**Fig 11:**
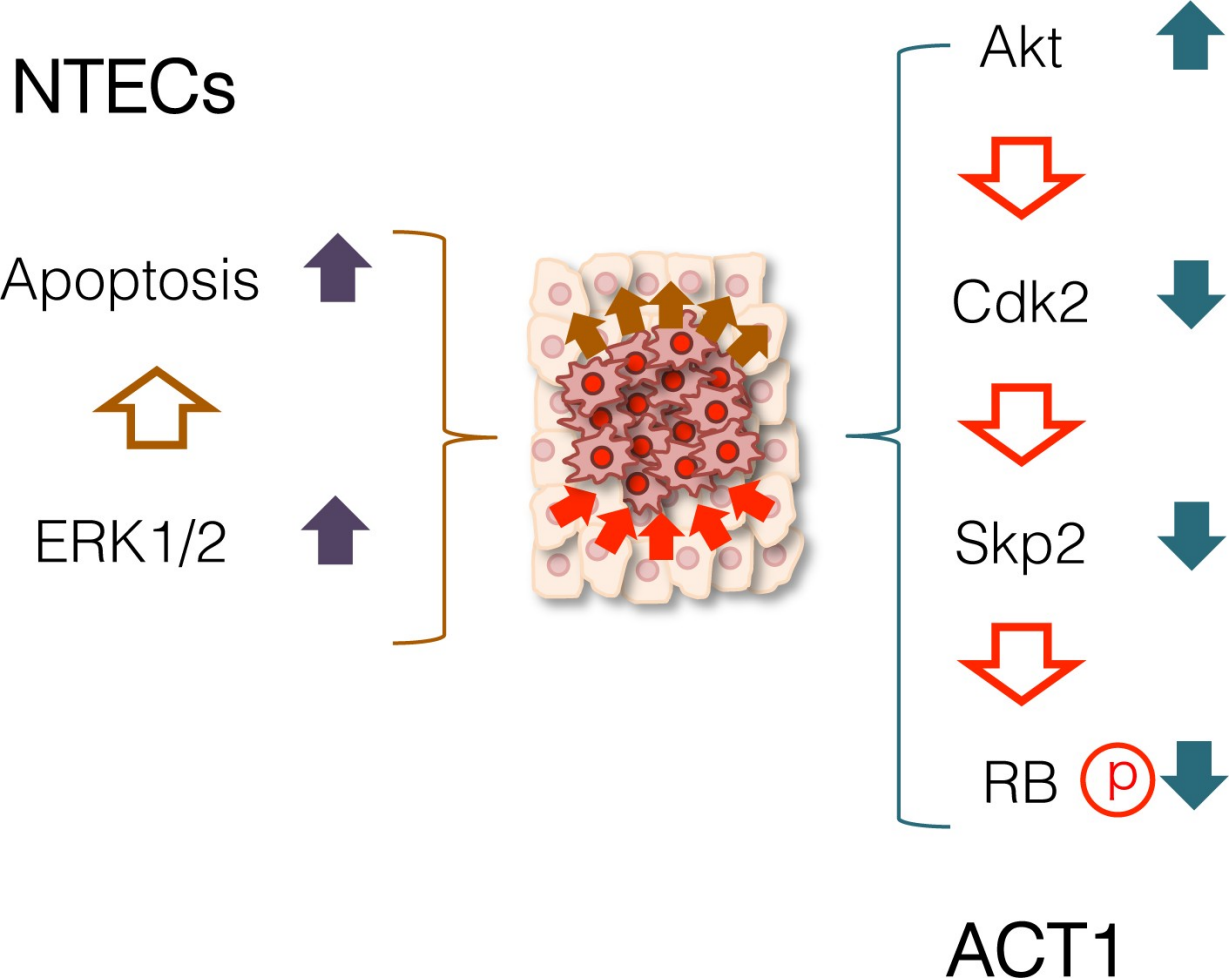
Summary of reciprocal stress response. Cell competition between anaplastic thyroid cancer cells and normal thyroid follicular epithelial cells provoked reciprocal stress response. In competed ACT1 cell clusters, activation of Akt resulted in dephosphorylation of RB, which gave rise to reduced growth. In contrast, competed NTECs, especially those neighboring ACT1 clusters, were forced to die by apoptosis via unscheduled activation of ERK1/2. Our results demonstrate that cell competition is a bi-directional phenomenon, thourgh which the initiation, propagation, and metastasis of tumors are hindered.

This study for the first time outlined the crucial role and possible mechanisms of the cell competition between anaplastic thyroid cancer cells and normal thyroid follicular epithelial cells. Comparison of ACT1 cluster size clearly indicated that the growth of ACT1 cells was significantly suppressed by NTECs (Fig 2). In fact, Ki-67 positivity was significantly reduced in ACT1 clusters, confirming that cell competition was able to suppress cancer cell growth (S3 Fig). Western blot analysis reveals that RB phosphorylation was significantly repressed accompanied by the up-regulation of p27^Kip1^ (Figs 7 and 8). Since p27^Kip1^ level is regulated through ubiquitin-dependent proteasome activity, we examined phosphorylation of Skp2, an E3 ligase catalyzing p27^Kip1^ ubiquitination [59], and found that it was reduced under competed condition (Fig 8B). We also observed up-regulation of Akt phosphorylation which facilitates Akt-dependent phosphorylation of Cdk2 and sequestering of the latter in the cytoplasm [60, 61]. These changes together with down-regulation of Cdk2 and Cyclin D could be involved in the retarded growth of competed ACT1 cells (Fig 11).

Besides ACT1 cell growth suppression, cell competition also affected the fate of NTECs manifested by the elimination of cells with contacting ACT1 clusters or located nearby to those. NTECs elimination was taking place through apoptosis accompanied by the region-specific activation of ERK1/2 (Fig 6), although the overall phosphorylation levels of ERK1/2 showed no change in bulk cultures (Fig 9). While activation of ERK1/2 is generally involved in cell proliferation, ERK activity has been also involved the induction of apoptosis [62]. Considering that no apoptosis in NTECs during the first days before the time-lapse analysis was started, this indicates that physical cell contact is required to induce NTECs elimination. Previously, it was demonstrated that mechanical stress through cell-to-cell contact was involved in cell competition [63, 64]. In fact, NTECs neighboring ACT1 cluster show increased cell anisotropy (S12D Fig), indicating that an increasing the number of NTECs competed with ACT1 clusters potentiates compression of NTECs at the border. This could cause apoptotic cell death in NTECs.

So far, the molecular nature of cell competition has been discussed extensively in Drosophila [21–25], and several driving factors have been identified including c-myc and p53 [26–29]. In fact, we confirmed augmented expression of c-myc in ACT1 cells (S13 Fig), however, it did not seem to make ACTs cells supercompetitor, since the growth of ACT1 is much slower than NTECs (S2 Fig). Also, differential expression of Scrib does not appear to be involved as there was no difference between NTECs and ACT1 (S13 Fig). The p53 gene is mutated in ACT1 cells, so that the p53 protein level is significantly stabilized (S13 Fig). Together with the mutation in the *N-ras* gene p53 deregulation could confer the ’winner’ phenotype [43], but ACT1 growth was indeed suppressed by the cell competition with NTECs, suggesting that driver mutations may play a different role in different cell context.

Recently, the suppressive effect of cell competition on liver cancer, which was mediated by the YAP induction in peritumoral hepatocytes, was reported [65]. The YAP as well as TAZ transcriptional coactivators are the downstream effectors of the Hippo signaling pathway, which plays critical roles in cell-to-cell contact, cell polarity, and fitness to the neighbors [66–68]. While several previous literatures have presented that the YAP1 is overexpressed in tumors including thyroid cancers [69, 70], the study clearly demonstrated that the YAP limitedly induced in normal hepatocytes neighboring tumor cells played suppressive role. In fact, the active YAP1 level was augmented in ACT1 cells, which was significantly down-regulated in co-culture (S14 Fig). Since high-density culture did not alter the YAP expression in ACT1 cells, cell competition with NTECs might affect the Hippo signaling pathway. Similar observation was reported by other study, confirming that bidirectional cell competition was indeed involved in cancer growth *in vivo* [71]. Thus, the down-regulation of YAP, which might be controlled by the Hippo signaling pathway activated through cell competition with NTECs, should be additional pathway that suppress ACT1 cell growth. The exact mechanism of the Hippo signal activation, and an involvement of Akt activation in this pathway need further investigations to confirm.

Previously, several reports demonstrated that cell growth rate and fitness *per se* may not drive cell competition as these are not an absolute common quality and does not determine the outcome of competition [27, 36, 72–75]. Our findings indicated that ATC1 cells have sensitivity to compaction and crowding, and the AKT activation could be a critical event to initiate the downstream cascade lead to RB dephosphorylation (Fig 11). The exact pathway of AKT activation still needs to be determined.

According to the results obtained by live-cell imaging (Fig 3), cell competition was initiated when the numbers of NTECs reached sufficient for cell compression. In order to confirm that continuous cell growth of NTECs is essential for cell competition, NTECs with sufficient cell number but terminated cell growth were examined (Fig 10). As we expected, reduction of ACT1 cluster sizes was significantly negated. We also found no apoptotic cell death in NTECs (S15 Fig). Furthermore, it is demonstrated that overall ERK1/2 phosphorylation is up-regulated upon irradiation (S16 Fig). While locally activated ERK1/2 seemed to be essential for apoptotic cell death of NTECs, the result suggested that not homogeneous activation but regional and accidental activation of ERK1/2 could be involved in region-specific apoptosis induction. Previously, it was reported that downregulation of ERK was involved in *Drosophila* [76], our results represented that the effect could be cell context-dependent.

It should be emphasized that even if the NTECs close to the ACT1 clusters were forced to die by apoptosis, the presence of NTECs surrounding cancer cells established suppressive cell competition to anaplastic cancer cells. Considering that thyroid follicles are the spheroidal structures formed by a monolayer of follicular cells, it is possible that similar cell competition could be taking place *in vivo*. Recently, accumulating evidences have demonstrated that cell competition is an essential component of tissue microenvironment, which is important not only during tissue development and aging but also in preventing cancer development and invasion [1–8]. Cellular heterogeneity of a tissue plays a key role since cancer cells may have different cell stiffness and different proliferation rates than normal cells. Although many of oncogenic mutations have been though to confer supercompetitor phenotype to cancer cells, normal counterparts exert intrinsic tumor-suppressive effects through cell competition activity, which was also reported by others and termed epithelial defence against cancer (EDAC) [77]. While anaplastic cancer is an aggressive form of thyroid cancer, our results indicated that cell competition therapy could be an option. Of importance, since our study showed that termination of growth of NTECs by radiation exposure abrogates suppressive competition properties of NTECs (Fig 10), it should be important to reduce toxicity of radiotherapy and chemotherapy to normal epithelial cells in order to preserve maximum EDAC.

In conclusion, we developed an *in vitro* cell competition system and demonstrated that the growth of anaplastic thyroid cancer cells was significantly retarded by normal thyroid follicular epithelial cells. Cell competition evoked stress response in cancer cells, which resulted in down-regulation of RB phosphorylation. Reciprocally, it induced stress response in normal cells, which gave rise to position-dependent induction of apoptosis. These results prove that cell competition is obviously a bidirectional phenomenon, in which competed cells are both affected each other. Since ERK1/2 and Akt, well-known pathways involved not only in anaplastic thyroid cancer but also in many other types of tumors, are critical components of cell competition (Fig 11), further studies on identifying target molecules that govern the struggle between cancer and normal cells could provide opportunities for conditioning the situation in favor of normal cells. Finally, it should be noted that our current experimental co-culture system has a potential limitation, as NTECs organize three-dimensional structure *in vivo*, and cell competition is not on a plastic surface but on the stroma with stromal cells, so that the future studies need to apply organoid culture, in which thyroid cancer cells and normal follicular cells are mixed together. Another limitation is that we only used one anaplastic thyroid cancer cell line, therefore, additional studies using different cancer cell clines should determine whether our observation is common phenomenon or not.

## Supporting information

S1 Fig Time-lapse analysis of GFP-NTECs crowding around ACT1 cell cluster. After ACT1 cell clusters were formed during 5 days’ culture, the GFP-H2B tagged NTECs were added, and the culture was chaced for 10 hours. A group of GFP-negative cells in the center of each image is ACT1 cell cluster. During the analysis, the images were captured at various time points as indicated. The bar in ‘0 hr’ indicates 40 μm.
(TIF)

S2 Fig Co-culture of ACT1 cell clusters and NTECs. ACT1 cell were cultured for 5 days before adding NTECs. NTECs grow and occupy space between ACT1 cell clusters, and become confluent by Day 5. The bar indicates 200 μm.
(TIF)

S3 Fig Growth of co-cultured ACT1 cell clusters with NTECs. ACT1 cells were cultured for 5 days until they formed small clusters before adding GFP-NTECs. Then, ACT1 clusters and GFP-NTECs were co-cultured for further 3 days, fixed with formaldehyde and stained with anti-Ki67 antibody (the secondary antibody is labelled with Alexa647, so that the green pseudo color was applied) and anti-53BP1 antibody (red fluorescence). (A) GFP-NTECs and ACT1 cluster showing 53BP1 staining (red). (B) GFP-NTECs and ACT1 cluster showing 53BP1 (red) and Ki-67 (green) staining. (C) Monoclutured ACT1 cluster showing 53BP1 (red) and Ki-67 (green) staining. The bar indicates 100 μm.
(TIF)

S4 Fig Time-lapse analysis showing apoptotic morphology of NTEC. ACT1 cells labeled with Qtracker 655 (red fluorescence) were incubated for 3 days before NTECs were added. Time-lapse imaging was started 48 hours after co-culture. During 75 minutes’ temporal observation, one NTEC, indicated by a white arrow head, show apoptotic cell morphology. The cell shows blebbing at time 5 min, and it becomes small round-shaped cell after 75 minutes.
(TIF)

S5 Fig Cell death in NTECs co-cultured with ACT1 cell clusters. ACT1 cells were cultured for 5 days until they formed small clusters before adding GFP-NTECs. Then, ACT1 clusters and GFP-NTECs were co-cultured for further 3 days, fixed with formaldehyde and stained with anti-vimentin antibody (the secondary antibody is labelled with Alexa647, so that the green and red pseudo color were applied) and anti-CDH1 antibody (red fluorescence). (A) GFP-NTECs and ACT1 cluster showing CDH1 staining in ACT1 cluster (red). (B) GFP-NTECs and ACT1 cluster showing CDH1 (red) and vimentin (green) staining. (C) GFP-NTECs and ACT1 cluster showing vimentin (red) staining, indicating that small round-shaped cells are vimentin positive. The bar indicates 100 μm.
(TIF)

S6 Fig DNA fragmentation in NTECs co-cultured with ACT1 cell clusters. ACT1 cells were cultured for 5 days until they formed small clusters before adding GFP-NTECs. Then, ACT1 clusters and GFP-NTECs were co-cultured for further 3 days, fixed with formaldehyde and stained with anti-phosphorylated H2AX antibody (red fluorescence). While some ACT1 cells in S phase are positive for phosphorylated H2AX, small round-shaped NTECs are strongly positive for phosphorylated H2AX, indicating the occurrence of DNA fragmentation. The bar indicates 100 μm.
(TIF)

S7 Fig Regional activation of ERK1/2 in NTECs co-cultured with ACT1 cell clusters. ACT1 cells were cultured for 5 days until they formed small clusters before adding GFP-NTECs. Then, ACT1 clusters and GFP-NTECs were co-cultured for further 3 days, fixed with formaldehyde and stained with anti-vimentin antibody (the secondary antibody is labelled with Alexa647, so that the green and red pseudo color were applied) and anti-phosphorylated ERK1/2 antibody (red fluorescence). (A) GFP-NTECs and ACT1 cluster showing ERK1/2 phosphorylation (red). While strong red fluorescence is observed in ACT1 cluster, some groups of NTECs show ERK1/2 phosphorylation. In addition, small round-shaped NTECs are positive for phosphorylated ERK1/2 antibody as indicated by white arrow heads. (B) GFP-NTECs and ACT1 cluster showing phosphorylated ERK1/2 (red) and vimentin (green) staining. (C) GFP-NTECs showing vimentin (red) staining. The bar indicates 100 μm.
(TIF)

S8 Fig Zoning of NTECs with phosphorylated ERK1/2. ACT1 cell clusters co-cultured with NTECs were stained with anti-vimentin antibody (green), and the ATC1 cluster is marked with a white dashed line (A), and NTECs with phosphorylated ERK1/2 signals were zoned according to their distance from the ACT1 cluster (B). The inner white dashed line indicates the border of ACT1 cell cluster. Dashed red line indicates 100 pixels from the cluster, orange 200 pixels, yellow 300 pixels, green 400 pixels, and cyan 500 pixels. The bar indicates 100 μm.
(TIF)

S9 Fig Differential cell collection. Co-cultures of ACT1 cells and NTECs were treated with 0.05% trypsin without PBS (A). After 2–3 minutes, detached NTECs were collected by gentle tapping the culture flasks (B). Then, cells were washed with PBS and re-treated with 0.05% trypsine for over 5 minutes to collect ACT1 cells (C). The bar indicates 200 μm.
(TIF)

S10 Fig Expression of senescence-associated β-galactosidase in terminally arrested NTECs. NTECs were exposed to 10 Gy of γ-rays before co-cultured with ACT1 cell clusters. Phase-contrast images of NETCs before (A) and 5 days after 10 Gy of γ-irradiation (B). NTECs were stained according to the protocol described in Materials and Methods.
(TIF)

S11 Fig Growth of co-cultureed ACT1 cell clusters with γ-irradiated NTECs. ACT1 cells were cultured for 5 days until they formed small clusters before adding GFP-NTECs. Then, ACT1 clusters and γ-irradiated GFP-NTECs were co-cultured for further 3 days, fixed with formaldehyde and stained with anti-Ki67 antibody (the secondary antibody is labelled with Alexa647, so that the green pseudo color was applied) and anti-53BP1 antibody (red fluorescence). (A) GFP-NTECs and ACT1 cluster showing 53BP1 staining (red). (B) GFP-NTECs and ACT1 cluster showing 53BP1 (red) and Ki-67 (green) staining. (C) Unirradiated GFP-NTECs and ACT1 cluster showing 53BP1 (red) and Ki-67 (green) staining. The bar indicates 100 μm.
(TIF)

S12 Fig Vimentin organization in NTECs co-cultured with ACT1 cell clusters. ACT1 cells were cultured for 5 days until they formed small clusters before adding GFP-NTECs. Then, ACT1 clusters and GFP-NTECs were co-cultured for further 3 days, fixed with formaldehyde and stained with anti-vimentin antibody (the secondary antibody is labelled with Alexa647, so that the green and red pseudo color were applied) and anti-CDH1 antibody (red fluorescence). (A) GFP-NTECs and ACT1 cluster showing CDH1 staining in ACT1 cluster (red). (B) GFP-NTECs and ACT1 cluster showing vimentin (green) staining. (C) Grayscale image of A. (D) Grayscale image of B. The bar indicates 100 μm.
(TIF)

S13 Fig Expression of proteins associated with cell competition. ACT1 cell clusters were co-cultured with GFP-NTECs for 5 days, fixed with formaldehyde, and stained with antibodies against c-Myc (red fluorescence) and p53 (green fluorescence). (A) Merged image. (B) Image showing c-Myc staining (red), which is overexpressed in ACT1 cluster. (C) Image showing p53 staining (green). The bar indicates 100 μm. (D) Total protein was extracted from both NTECs and ACT1 cells cultured either alone (NTEC or ACT1 only, respectively) or from co-cultures (NTEC or ACT1 competed, respectively) and subjected to western blot analysis with indicated antibodies.
(TIF)

S14 Fig Down-regulation of the YAP expression through cell competition. ACT1 cells were cultured for 5 days until they formed small clusters before adding NTECs. Then, ACT1 clusters and NTECs were co-cultured for further 3 days, fixed with formaldehyde and stained with anti-vimentin antibody (the secondary antibody is labelled with Alexa647, so that the green pseudo color was applied) and anti-active YAP1 antibody (red fluorescence). Exponentially growing NTECs (A), ACT1 cluster (B), confluent NTECs (C), and confluent ACT1 cells (D) show vimentin (green) and active YAP1 (red) expression. ACT1 cluster co-cultured with NTECs show decreased active YAP1 expression. The bar in (A) indicates 40 μm.
(TIF)

S15 Fig No apoptotic cell death and activation of ERK1/2 in 10 Gy-irradiated NTECs co-cultured with ACT1 cell clusters. ACT1 cells were cultured for 5 days until they formed small clusters before adding 10 Gy-irradiated GFP-NTECs. Then, ACT1 clusters and GFP-NTECs were co-cultured for further 3 days, fixed with formaldehyde and stained with anti-vimentin antibody (the secondary antibody is labelled with Alexa647, so that the green and red pseudo color were applied) and anti-phosphorylated ERK1/2 antibody (red fluorescence). (A) GFP-NTECs and ACT1 cluster showing ERK1/2 phosphorylation (red). (B) GFP-NTECs and ACT1 cluster showing phosphorylated ERK1/2 (red) and vimentin (green) staining. (C) GFP-NTECs and ACT1 cluster showing vimentin (red) staining. No apoptotic small round-shaped cells are detected. The bar indicates 100 μm.
(TIF)

S16 Fig Detection of phosphorylation of ERK1/2. ACT1 cell clusters were co-cultured with 10 Gy-irradiated GFP-NTECs for 24 hours, fixed with formaldehyde, and stained with anti-phosphorylated ERK1/2 antibody (red). (A) ACT1 cluster was co-cultured with unirradiated NTECs. (B) ACT1 cluster was co-cultured with 10 Gy-irradiated NTECs, showing ubiquitous phosphorylation of ERK1/2 in 10 Gy-irradiated NTECs. The bar indicates 100 μm.
(TIF)

S1 Movie Time-lapse analysis showing apoptotic morphology in competed NTECs. ACT1 cells labeled with Qtracker 655 (red fluorescence) were incubated for 3 days before NTECs were added. Time-lapse imaging was started 48 hours after co-culture. During 58 hours’ observation, NTECs neighboring ACT1 clusters show apoptotic cell morphology.
(MP4)

S2 Movie Time-lapse analysis demonstrates Caspase 3/7 activation in NTECs neighboring ACT1 cluster. ACT1 cells were incubated for 3 days before NTECs were added. Time-lapse imaging was started 48 hours after co-culture at which time point CellEvent Caspase-3/7 Green detection reagent was added and incubated for 30 min. During 24 hours’ observation, NTECs with CellEvent Caspase-3/7 green fluorescence are detectable.
(MP4)

S1 Raw images (PDF)
