# Supplementary figures and images for: Cell competition between anaplastic thyroid cancer and normal thyroid follicular cells exerts reciprocal stress response defining tumor suppressive effects of normal epithelial tissue
Source: PLoS One. 2021 Apr 1;16(4):e0249059. doi: 10.1371/journal.pone.0249059 (PMC8016217; doi:10.1371/journal.pone.0249059)

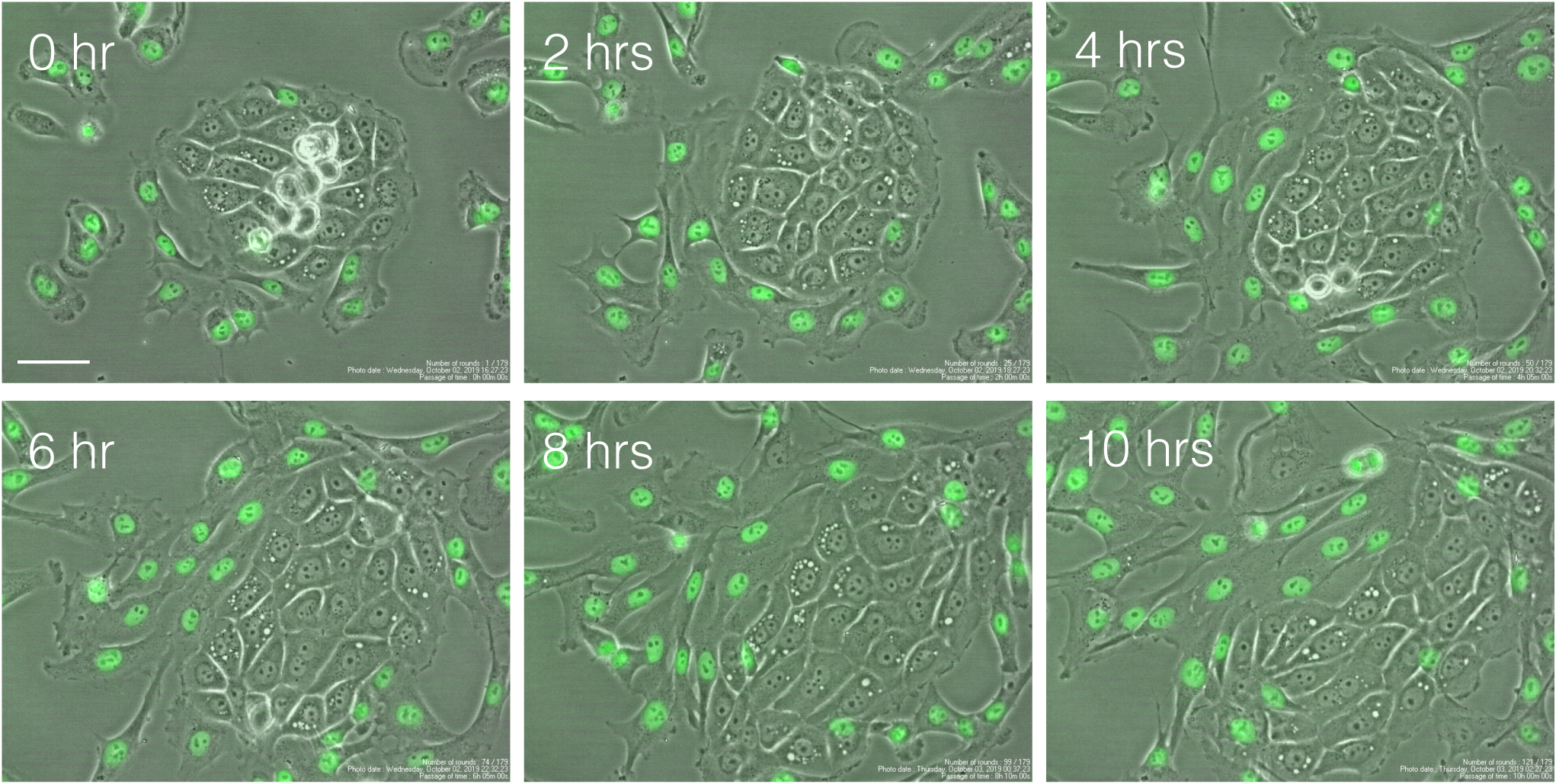

Supplement: S1 Fig — After ACT1 cell clusters were formed during 5 days’ culture, the GFP-H2B tagged NTECs were added, and the culture was chaced for 10 hours. A group of GFP-negative cells in the center of each image is ACT1 cell cluster. During the analysis, the images were captured at various time points as indicated. The bar in ‘0 hr’ indicates 40 μm. (TIF) [file pone.0249059.s001.tif]

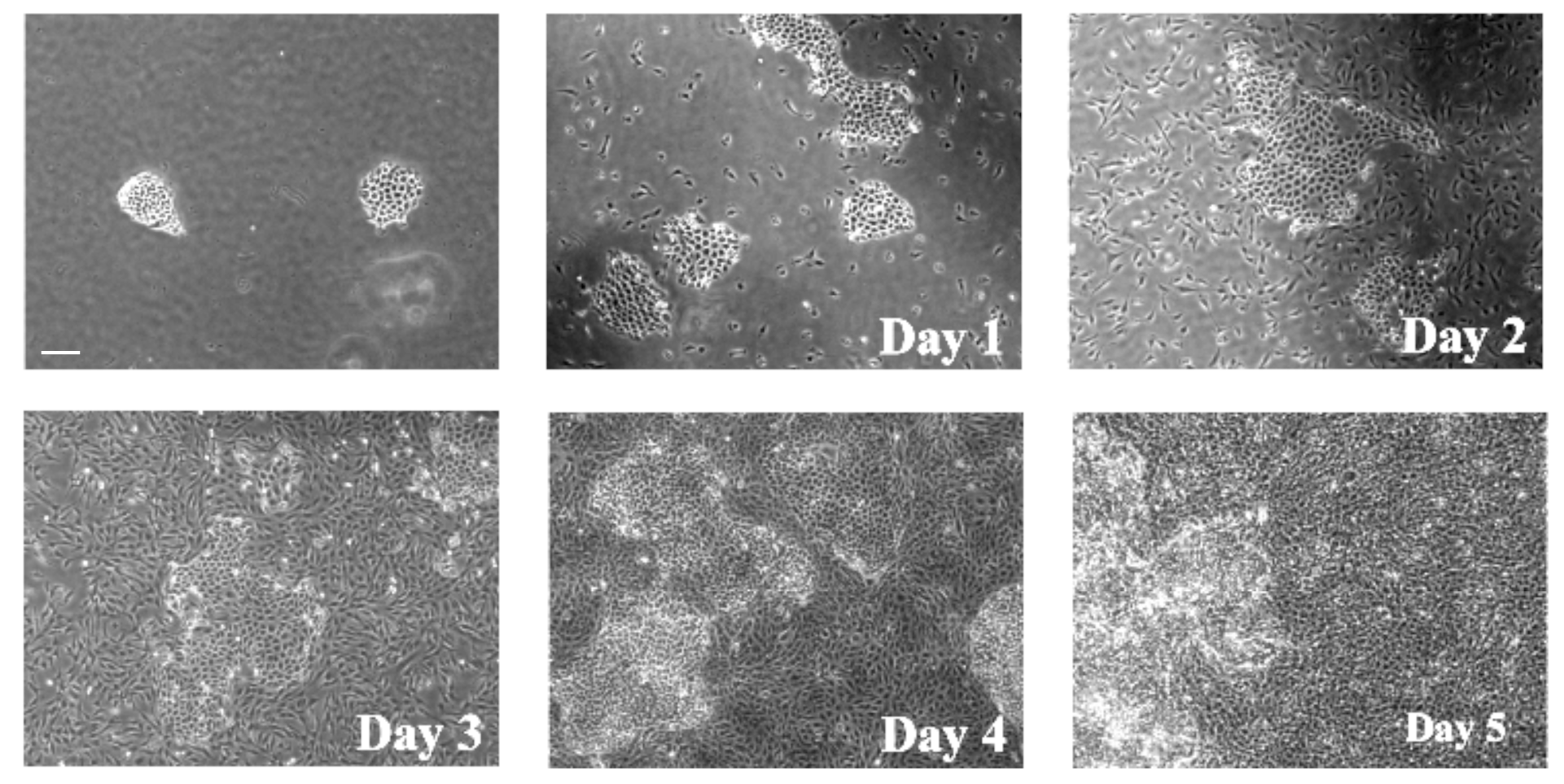

Supplement: S2 Fig — ACT1 cell were cultured for 5 days before adding NTECs. NTECs grow and occupy space between ACT1 cell clusters, and become confluent by Day 5. The bar indicates 200 μm. (TIF) [file pone.0249059.s002.tif]

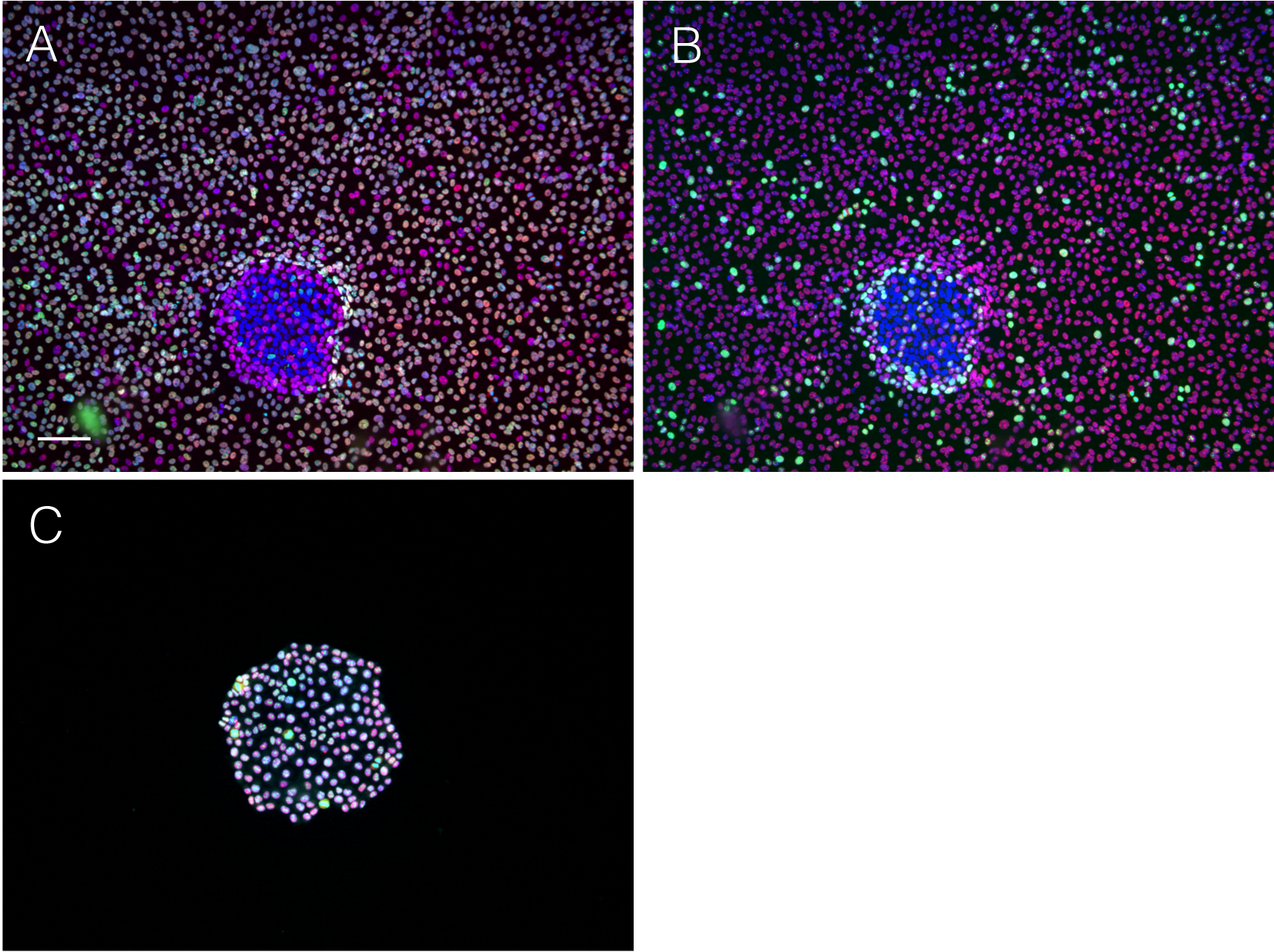

Supplement: S3 Fig — ACT1 cells were cultured for 5 days until they formed small clusters before adding GFP-NTECs. Then, ACT1 clusters and GFP-NTECs were co-cultured for further 3 days, fixed with formaldehyde and stained with anti-Ki67 antibody (the secondary antibody is labelled with Alexa647, so that the green pseudo color was applied) and anti-53BP1 antibody (red fluorescence). (A) GFP-NTECs and ACT1 cluster showing 53BP1 staining (red). (B) GFP-NTECs and ACT1 cluster showing 53BP1 (red) and Ki-67 (green) staining. (C) Monoclutured ACT1 cluster showing 53BP1 (red) and Ki-67 (green) staining. The bar indicates 100 μm. (TIF) [file pone.0249059.s003.tif]

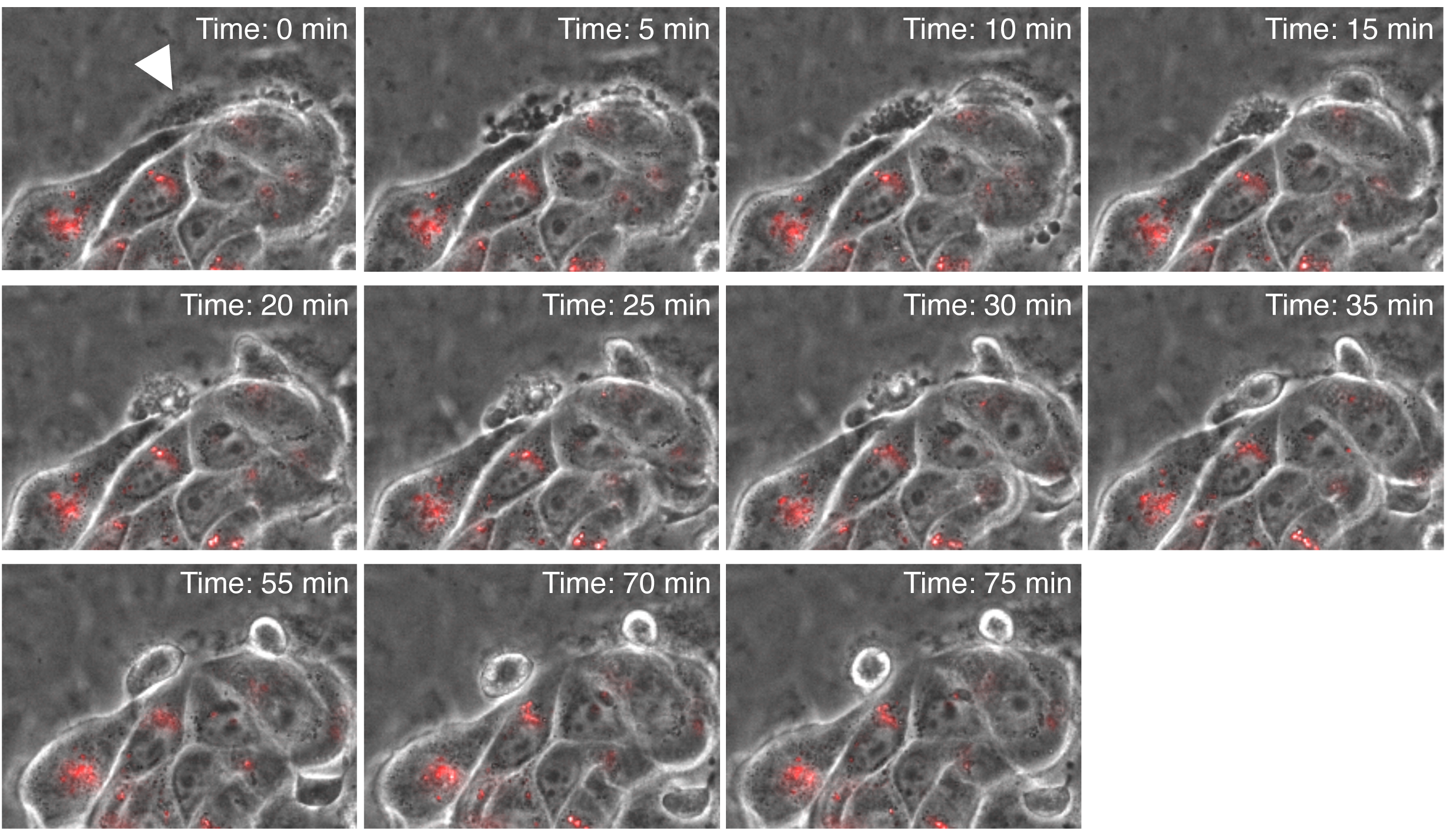

Supplement: S4 Fig — ACT1 cells labeled with Qtracker 655 (red fluorescence) were incubated for 3 days before NTECs were added. Time-lapse imaging was started 48 hours after co-culture. During 75 minutes’ temporal observation, one NTEC, indicated by a white arrow head, show apoptotic cell morphology. The cell shows blebbing at time 5 min, and it becomes small round-shaped cell after 75 minutes. (TIF) [file pone.0249059.s004.tif]

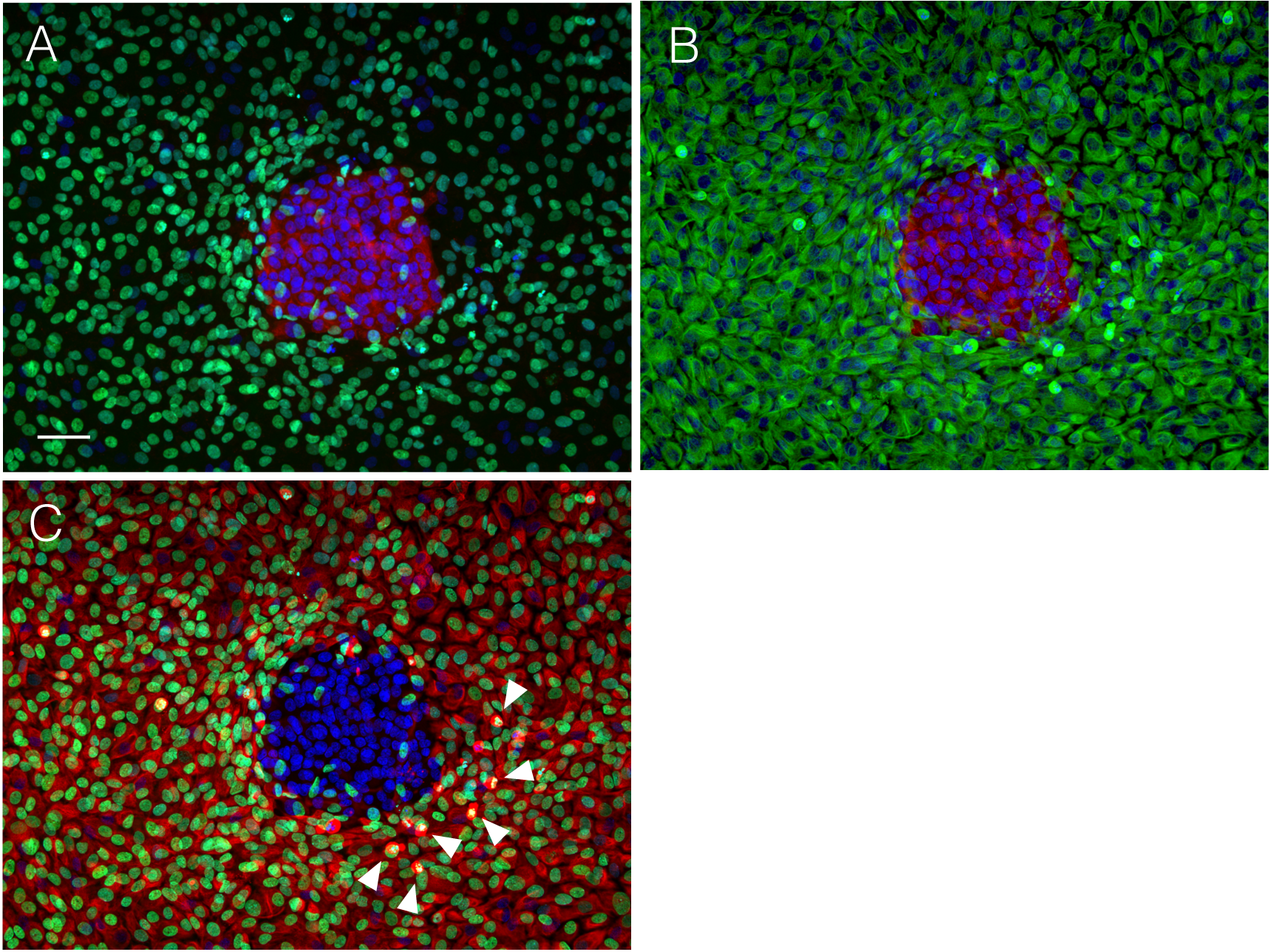

Supplement: S5 Fig — ACT1 cells were cultured for 5 days until they formed small clusters before adding GFP-NTECs. Then, ACT1 clusters and GFP-NTECs were co-cultured for further 3 days, fixed with formaldehyde and stained with anti-vimentin antibody (the secondary antibody is labelled with Alexa647, so that the green and red pseudo color were applied) and anti-CDH1 antibody (red fluorescence). (A) GFP-NTECs and ACT1 cluster showing CDH1 staining in ACT1 cluster (red). (B) GFP-NTECs and ACT1 cluster showing CDH1 (red) and vimentin (green) staining. (C) GFP-NTECs and ACT1 cluster showing vimentin (red) staining, indicating that small round-shaped cells are vimentin positive. The bar indicates 100 μm. (TIF) [file pone.0249059.s005.tif]

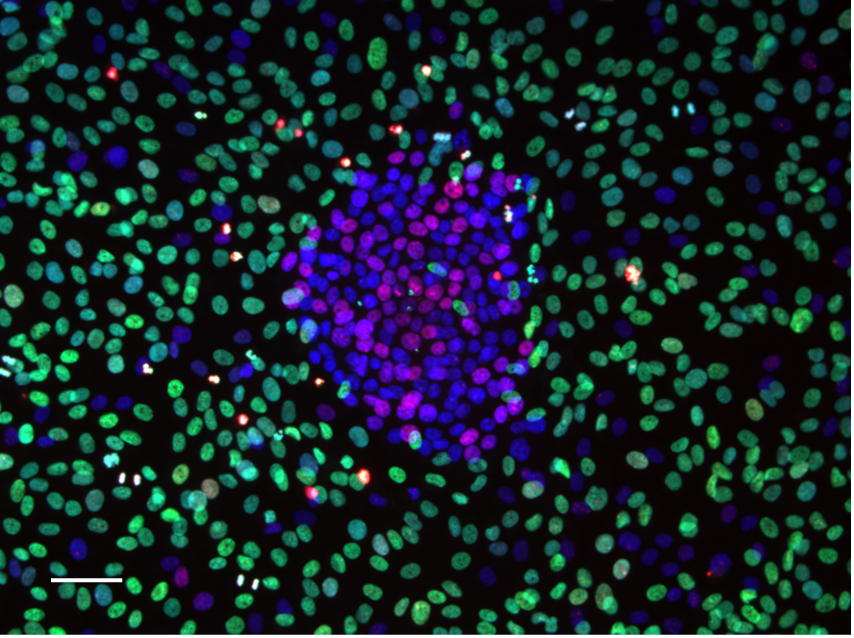

Supplement: S6 Fig — ACT1 cells were cultured for 5 days until they formed small clusters before adding GFP-NTECs. Then, ACT1 clusters and GFP-NTECs were co-cultured for further 3 days, fixed with formaldehyde and stained with anti-phosphorylated H2AX antibody (red fluorescence). While some ACT1 cells in S phase are positive for phosphorylated H2AX, small round-shaped NTECs are strongly positive for phosphorylated H2AX, indicating the occurrence of DNA fragmentation. The bar indicates 100 μm. (TIF) [file pone.0249059.s006.tif]

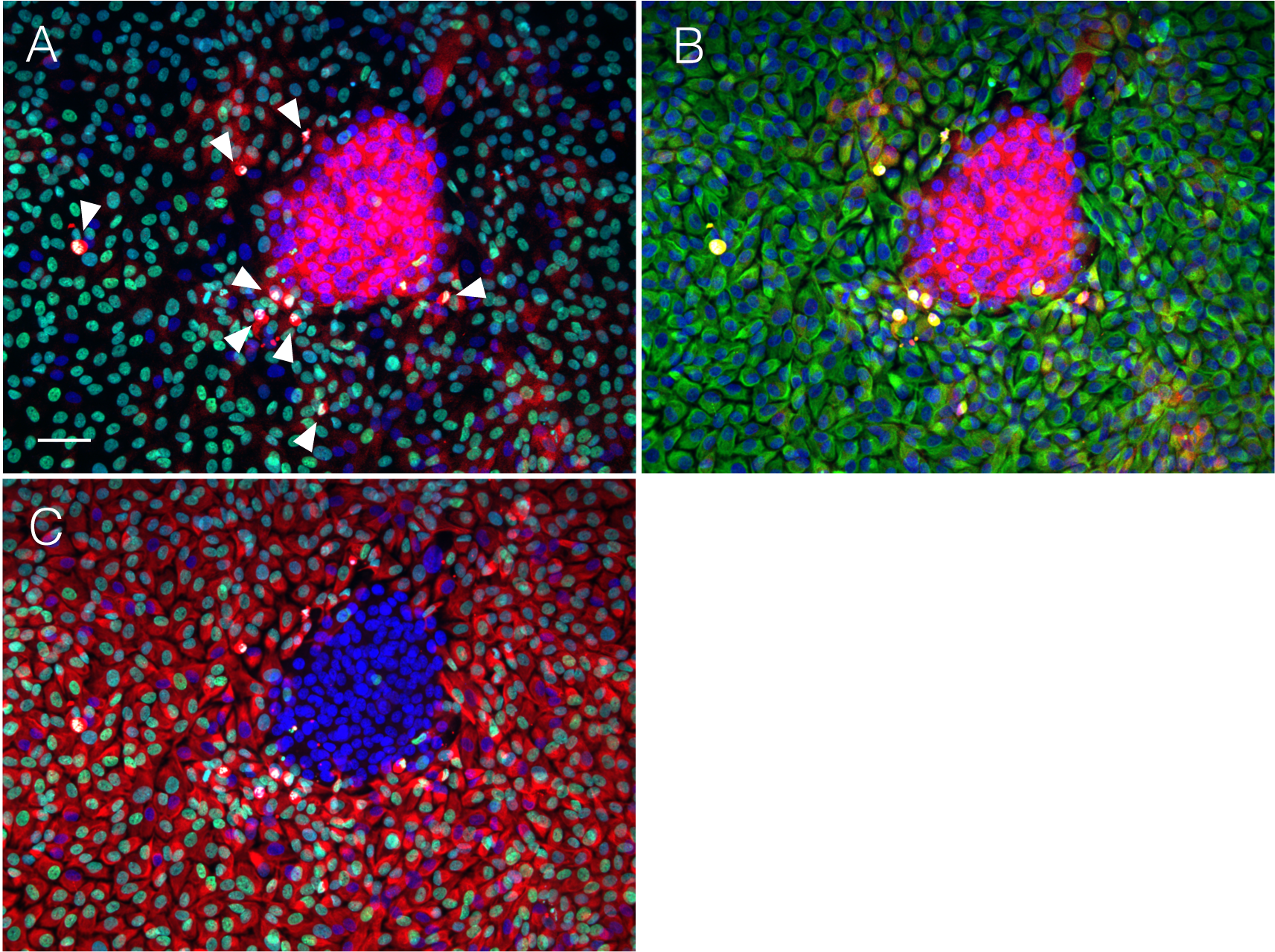

Supplement: S7 Fig — ACT1 cells were cultured for 5 days until they formed small clusters before adding GFP-NTECs. Then, ACT1 clusters and GFP-NTECs were co-cultured for further 3 days, fixed with formaldehyde and stained with anti-vimentin antibody (the secondary antibody is labelled with Alexa647, so that the green and red pseudo color were applied) and anti-phosphorylated ERK1/2 antibody (red fluorescence). (A) GFP-NTECs and ACT1 cluster showing ERK1/2 phosphorylation (red). While strong red fluorescence is observed in ACT1 cluster, some groups of NTECs show ERK1/2 phosphorylation. In addition, small round-shaped NTECs are positive for phosphorylated ERK1/2 antibody as indicated by white arrow heads. (B) GFP-NTECs and ACT1 cluster showing phosphorylated ERK1/2 (red) and vimentin (green) staining. (C) GFP-NTECs showing vimentin (red) staining. The bar indicates 100 μm. (TIF) [file pone.0249059.s007.tif]

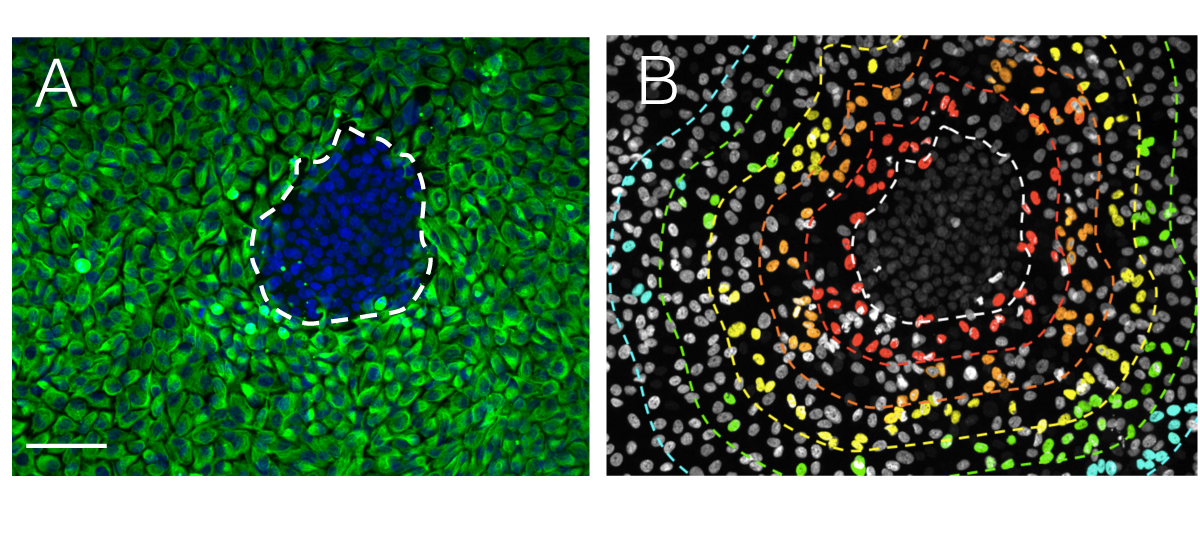

Supplement: S8 Fig — ACT1 cell clusters co-cultured with NTECs were stained with anti-vimentin antibody (green), and the ATC1 cluster is marked with a white dashed line (A), and NTECs with phosphorylated ERK1/2 signals were zoned according to their distance from the ACT1 cluster (B). The inner white dashed line indicates the border of ACT1 cell cluster. Dashed red line indicates 100 pixels from the cluster, orange 200 pixels, yellow 300 pixels, green 400 pixels, and cyan 500 pixels. The bar indicates 100 μm. (TIF) [file pone.0249059.s008.tif]

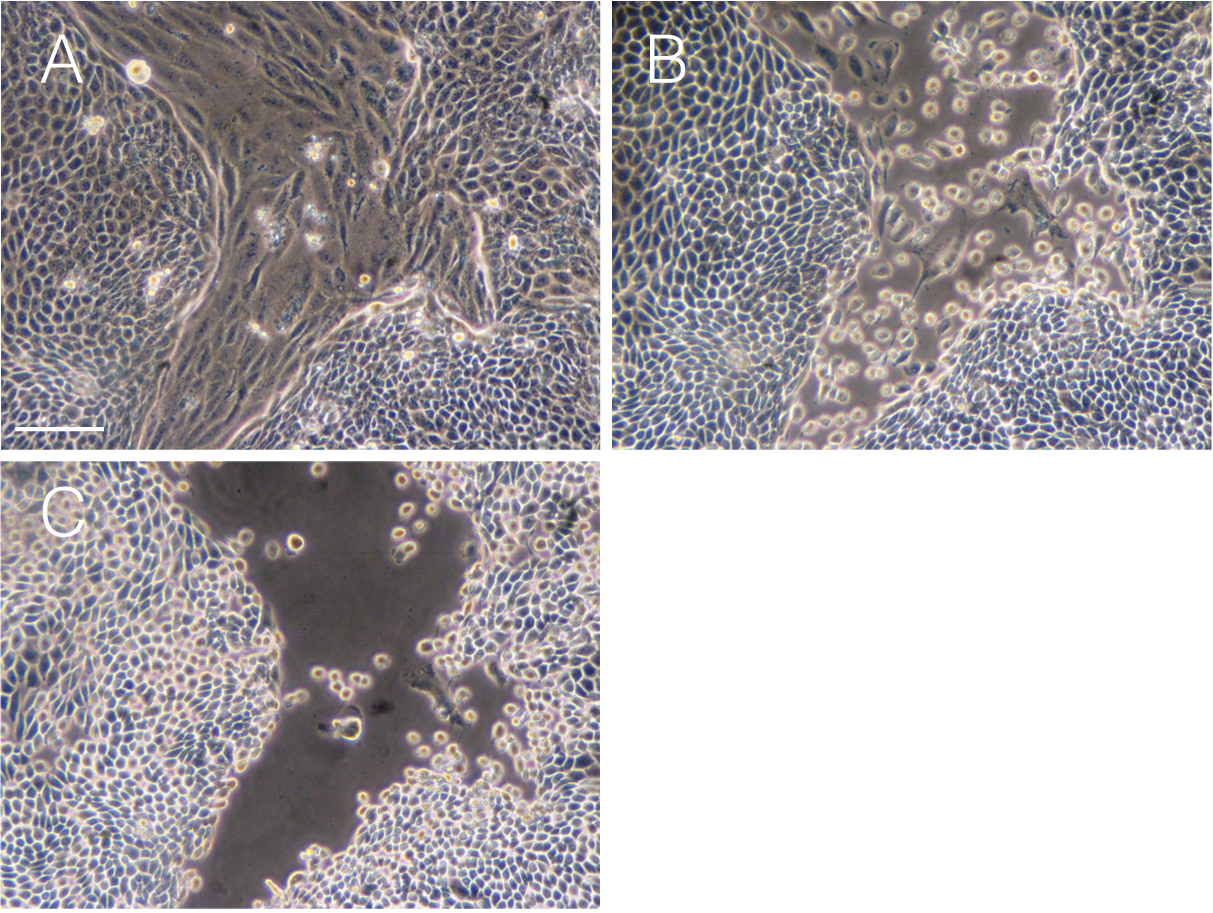

Supplement: S9 Fig — Co-cultures of ACT1 cells and NTECs were treated with 0.05% trypsin without PBS (A). After 2–3 minutes, detached NTECs were collected by gentle tapping the culture flasks (B). Then, cells were washed with PBS and re-treated with 0.05% trypsine for over 5 minutes to collect ACT1 cells (C). The bar indicates 200 μm. (TIF) [file pone.0249059.s009.tif]

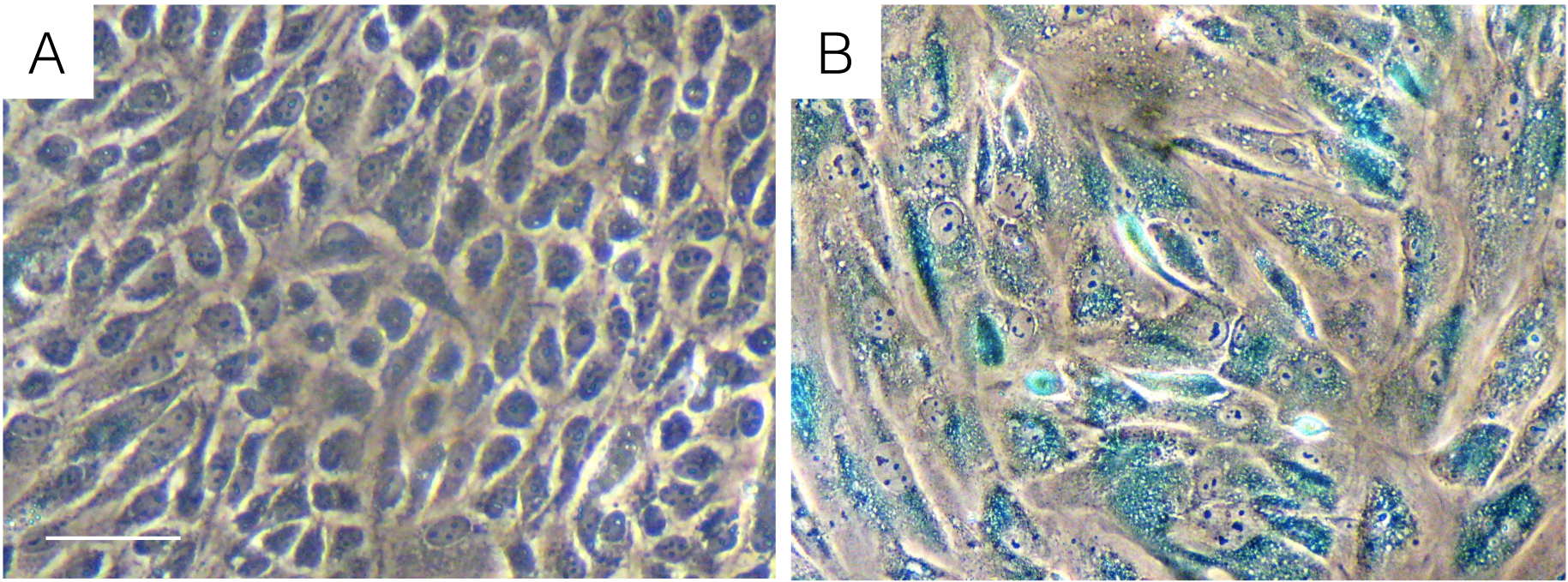

Supplement: S10 Fig — NTECs were exposed to 10 Gy of γ-rays before co-cultured with ACT1 cell clusters. Phase-contrast images of NETCs before (A) and 5 days after 10 Gy of γ-irradiation (B). NTECs were stained according to the protocol described in Materials and Methods. (TIF) [file pone.0249059.s010.tif]

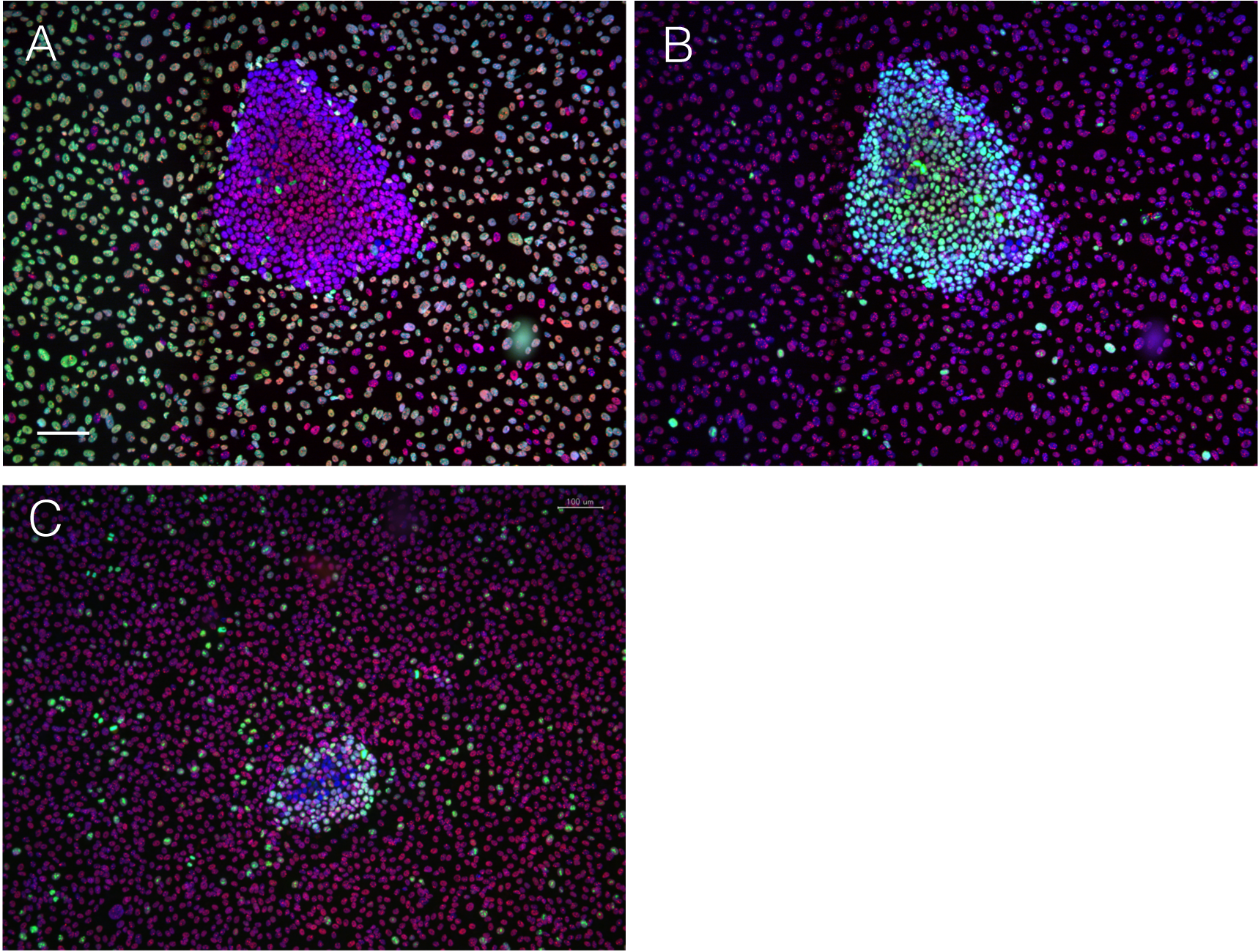

Supplement: S11 Fig — ACT1 cells were cultured for 5 days until they formed small clusters before adding GFP-NTECs. Then, ACT1 clusters and γ-irradiated GFP-NTECs were co-cultured for further 3 days, fixed with formaldehyde and stained with anti-Ki67 antibody (the secondary antibody is labelled with Alexa647, so that the green pseudo color was applied) and anti-53BP1 antibody (red fluorescence). (A) GFP-NTECs and ACT1 cluster showing 53BP1 staining (red). (B) GFP-NTECs and ACT1 cluster showing 53BP1 (red) and Ki-67 (green) staining. (C) Unirradiated GFP-NTECs and ACT1 cluster showing 53BP1 (red) and Ki-67 (green) staining. The bar indicates 100 μm. (TIF) [file pone.0249059.s011.tif]

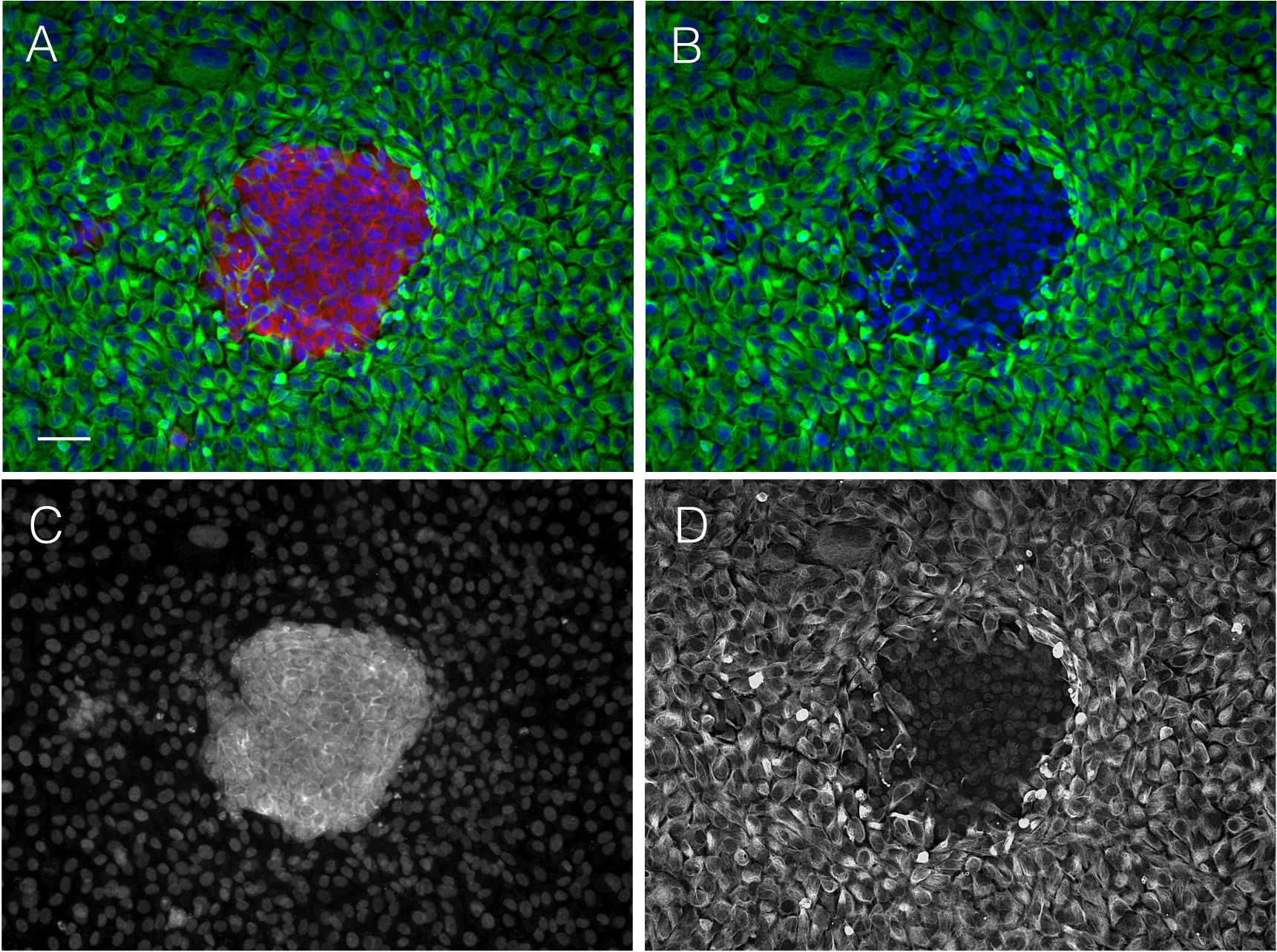

Supplement: S12 Fig — ACT1 cells were cultured for 5 days until they formed small clusters before adding GFP-NTECs. Then, ACT1 clusters and GFP-NTECs were co-cultured for further 3 days, fixed with formaldehyde and stained with anti-vimentin antibody (the secondary antibody is labelled with Alexa647, so that the green and red pseudo color were applied) and anti-CDH1 antibody (red fluorescence). (A) GFP-NTECs and ACT1 cluster showing CDH1 staining in ACT1 cluster (red). (B) GFP-NTECs and ACT1 cluster showing vimentin (green) staining. (C) Grayscale image of A. (D) Grayscale image of B. The bar indicates 100 μm. (TIF) [file pone.0249059.s012.tif]

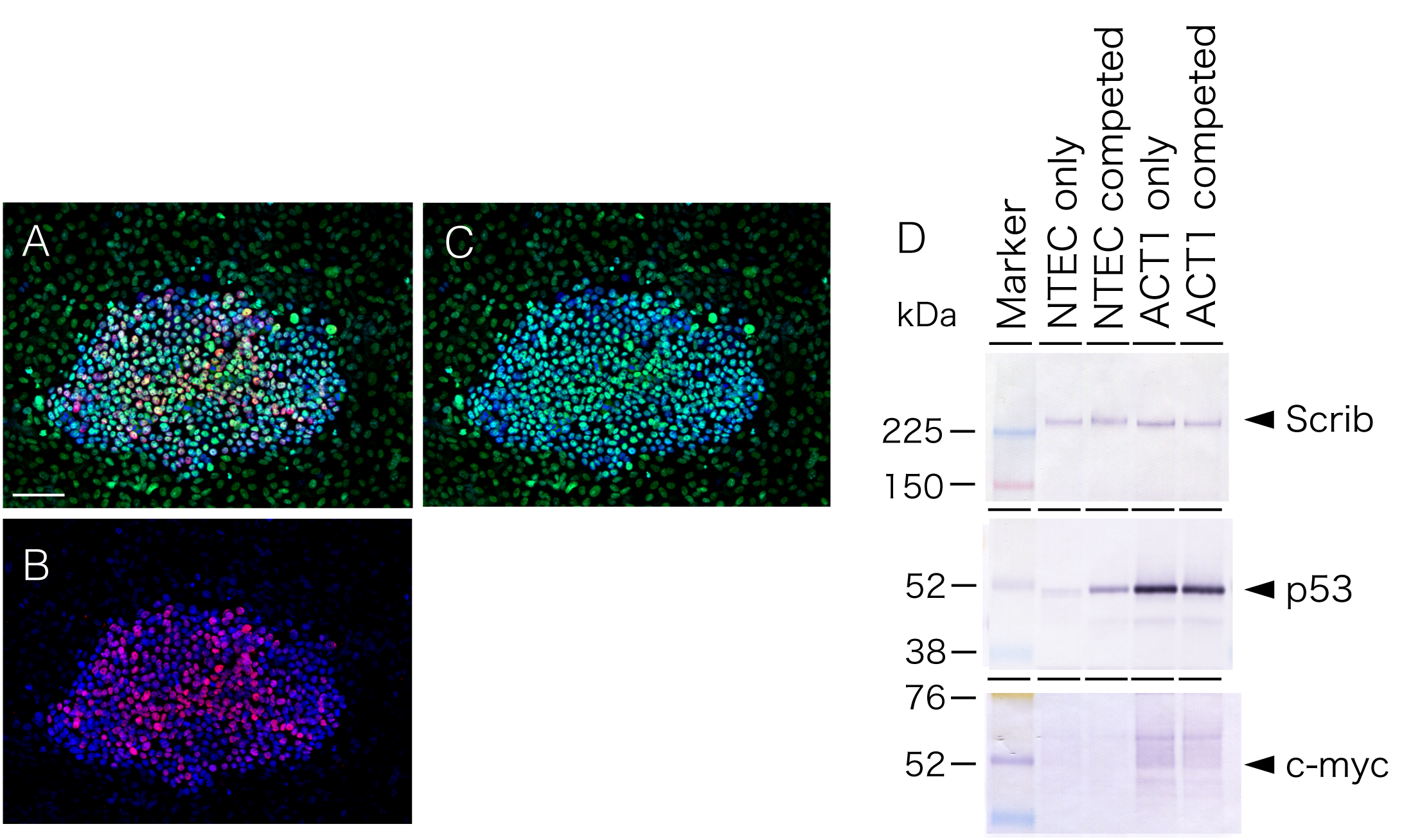

Supplement: S13 Fig — ACT1 cell clusters were co-cultured with GFP-NTECs for 5 days, fixed with formaldehyde, and stained with antibodies against c-Myc (red fluorescence) and p53 (green fluorescence). (A) Merged image. (B) Image showing c-Myc staining (red), which is overexpressed in ACT1 cluster. (C) Image showing p53 staining (green). The bar indicates 100 μm. (D) Total protein was extracted from both NTECs and ACT1 cells cultured either alone (NTEC or ACT1 only, respectively) or from co-cultures (NTEC or ACT1 competed, respectively) and subjected to western blot analysis with indicated antibodies. (TIF) [file pone.0249059.s013.tif]

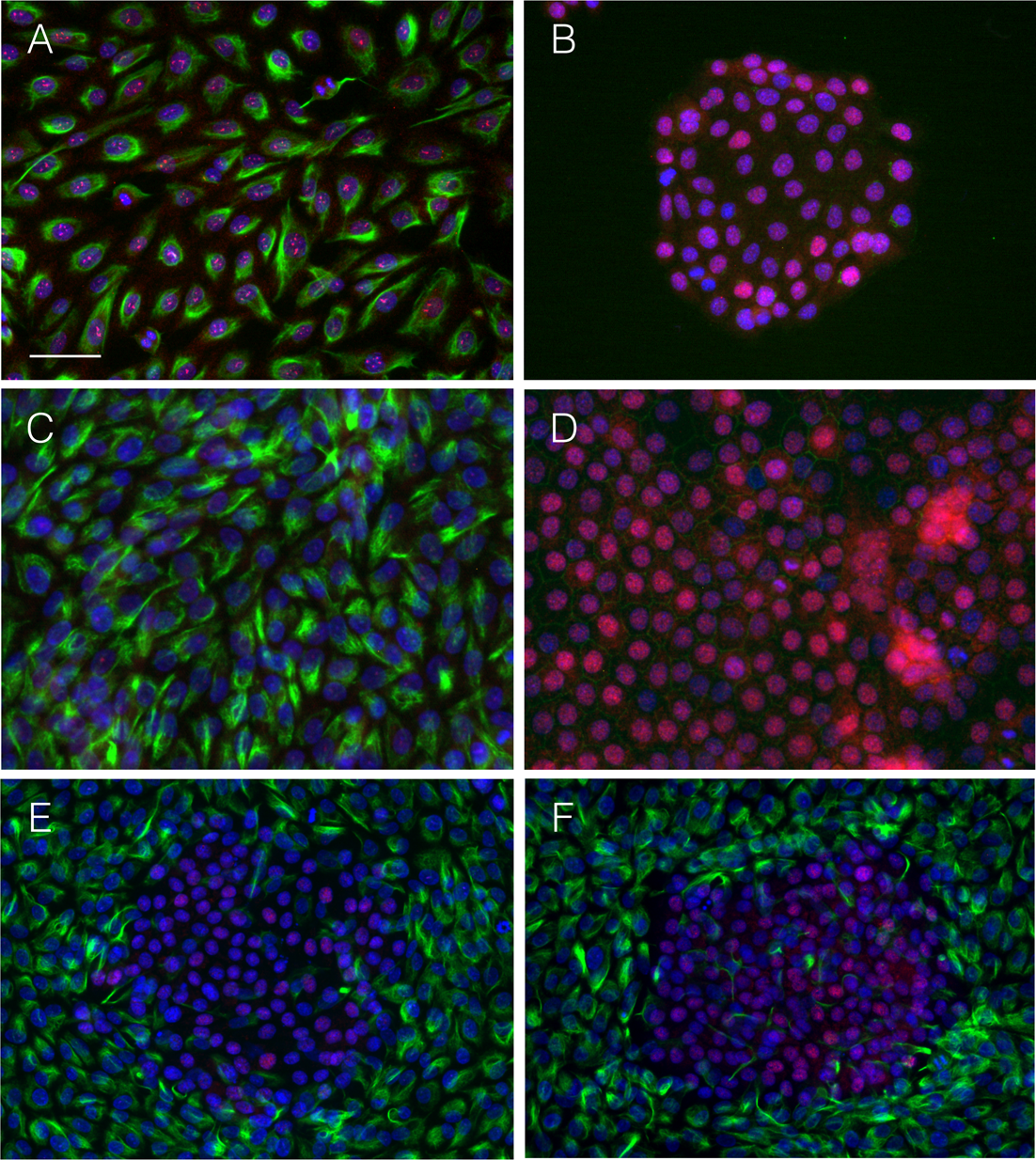

Supplement: S14 Fig — ACT1 cells were cultured for 5 days until they formed small clusters before adding NTECs. Then, ACT1 clusters and NTECs were co-cultured for further 3 days, fixed with formaldehyde and stained with anti-vimentin antibody (the secondary antibody is labelled with Alexa647, so that the green pseudo color was applied) and anti-active YAP1 antibody (red fluorescence). Exponentially growing NTECs (A), ACT1 cluster (B), confluent NTECs (C), and confluent ACT1 cells (D) show vimentin (green) and active YAP1 (red) expression. ACT1 cluster co-cultured with NTECs show decreased active YAP1 expression. The bar in (A) indicates 40 μm. (TIF) [file pone.0249059.s014.tif]

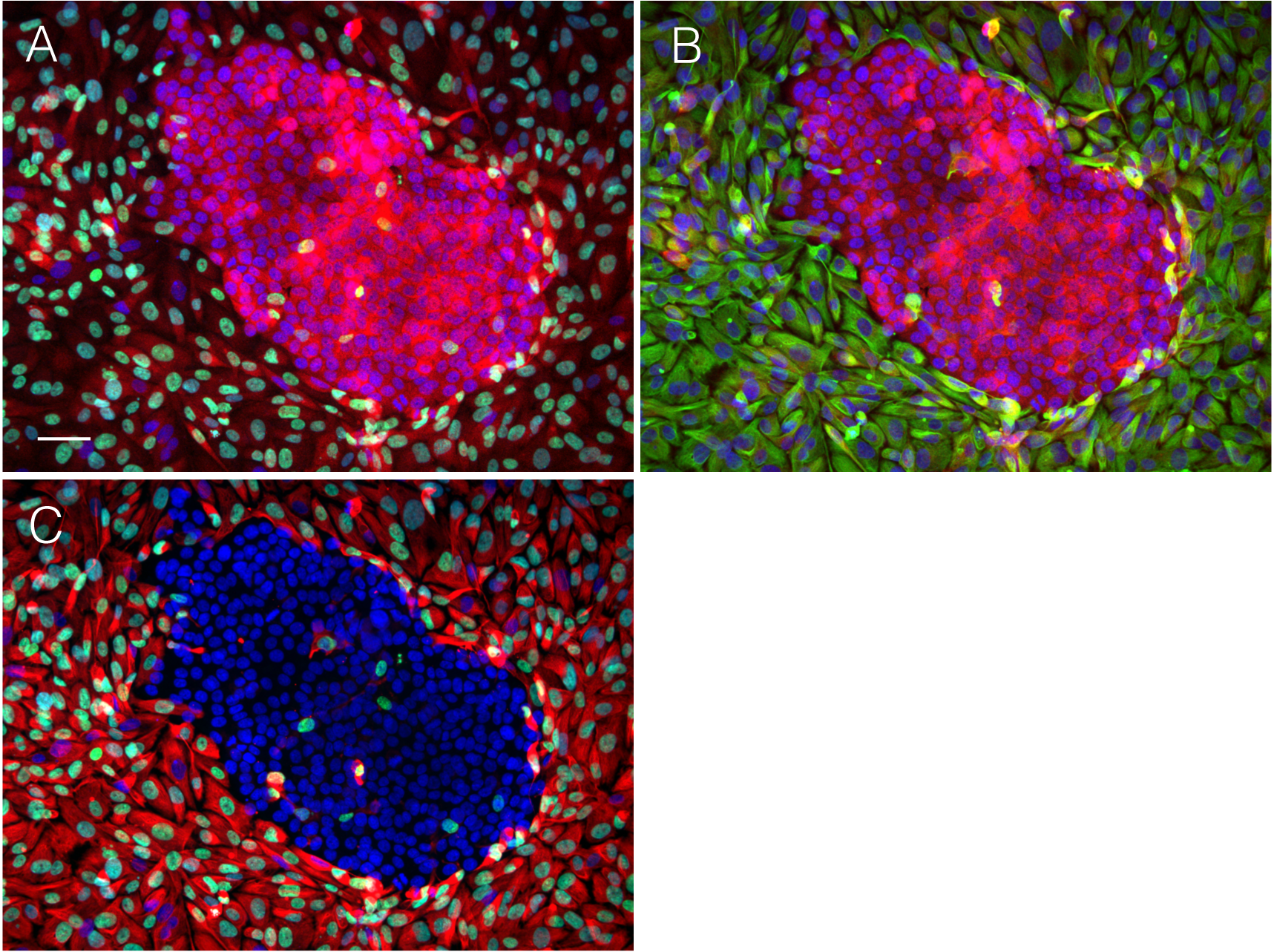

Supplement: S15 Fig — ACT1 cells were cultured for 5 days until they formed small clusters before adding 10 Gy-irradiated GFP-NTECs. Then, ACT1 clusters and GFP-NTECs were co-cultured for further 3 days, fixed with formaldehyde and stained with anti-vimentin antibody (the secondary antibody is labelled with Alexa647, so that the green and red pseudo color were applied) and anti-phosphorylated ERK1/2 antibody (red fluorescence). (A) GFP-NTECs and ACT1 cluster showing ERK1/2 phosphorylation (red). (B) GFP-NTECs and ACT1 cluster showing phosphorylated ERK1/2 (red) and vimentin (green) staining. (C) GFP-NTECs and ACT1 cluster showing vimentin (red) staining. No apoptotic small round-shaped cells are detected. The bar indicates 100 μm. (TIF) [file pone.0249059.s015.tif]

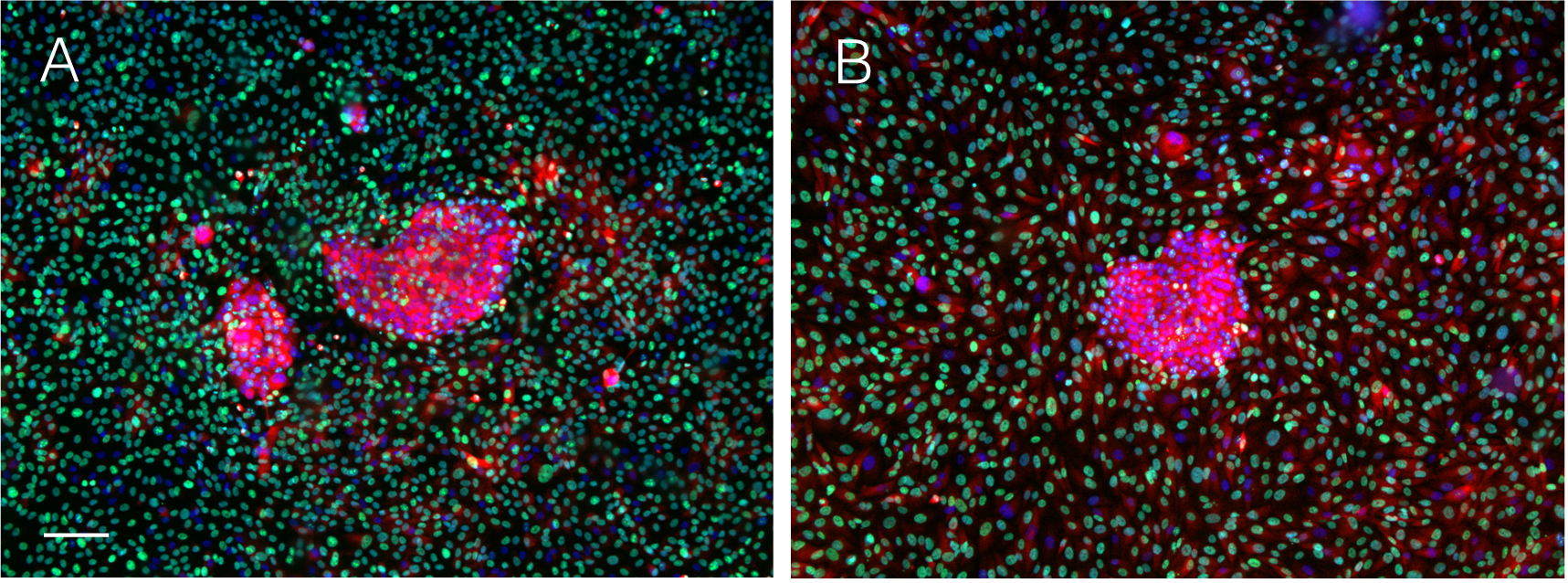

Supplement: S16 Fig — ACT1 cell clusters were co-cultured with 10 Gy-irradiated GFP-NTECs for 24 hours, fixed with formaldehyde, and stained with anti-phosphorylated ERK1/2 antibody (red). (A) ACT1 cluster was co-cultured with unirradiated NTECs. (B) ACT1 cluster was co-cultured with 10 Gy-irradiated NTECs, showing ubiquitous phosphorylation of ERK1/2 in 10 Gy-irradiated NTECs. The bar indicates 100 μm. (TIF) [file pone.0249059.s016.tif]
