# Supplementary material for: Cell competition between anaplastic thyroid cancer and normal thyroid follicular cells exerts reciprocal stress response defining tumor suppressive effects of normal epithelial tissue
Source: PLoS One. 2021 Apr 1;16(4):e0249059. doi: 10.1371/journal.pone.0249059 (PMC8016217; doi:10.1371/journal.pone.0249059)
Supplement: S1 Raw images — (PDF) [file pone.0249059.s019.pdf]

These images are for Figure 7

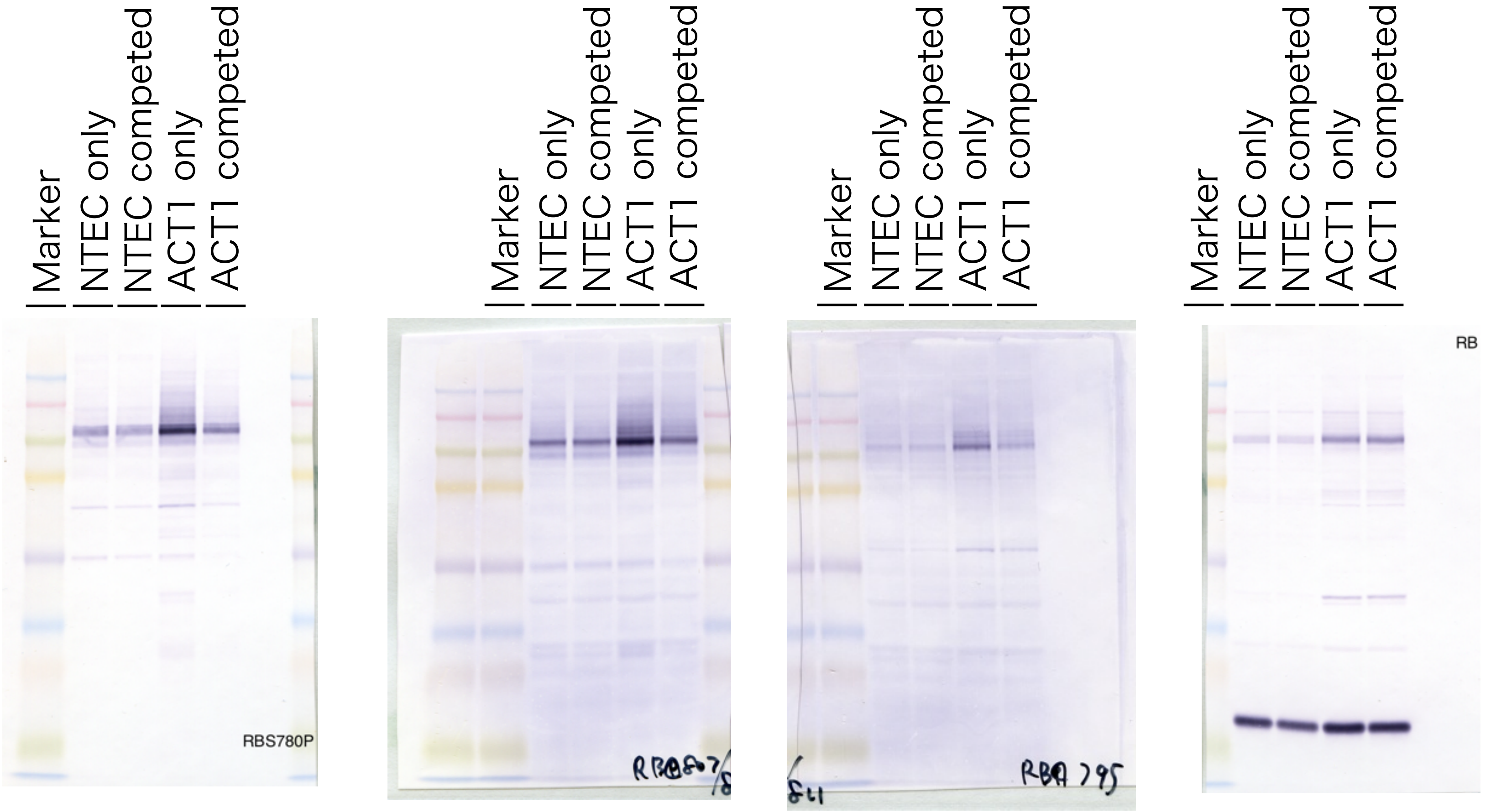

RB S780 ⓟ

RB S807/811 ⓟ

RB S795 ⓟ

RB

These images are for Figure 7

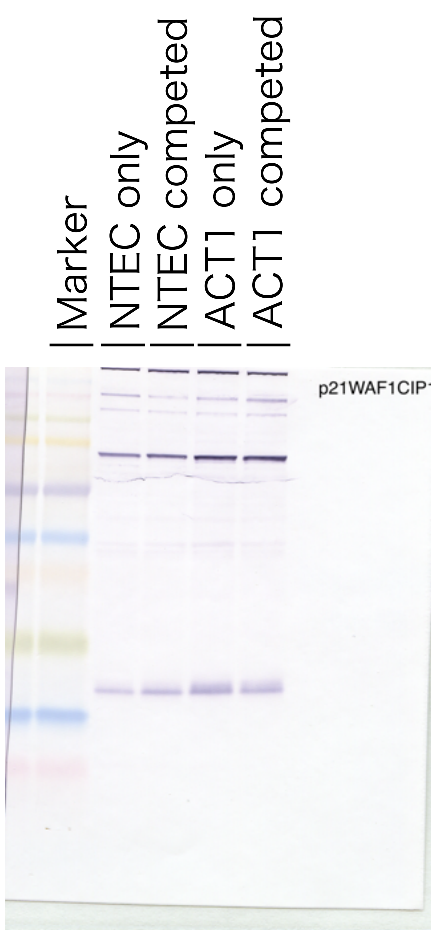

p21$^{WAF1/Cip1}$

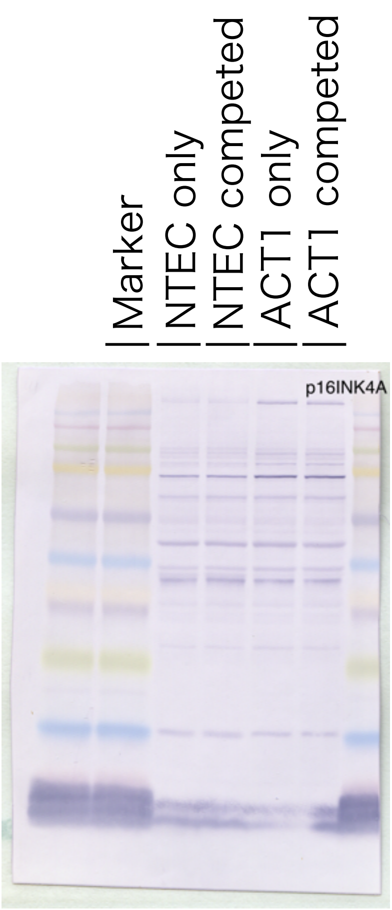

p16^{INK4A}

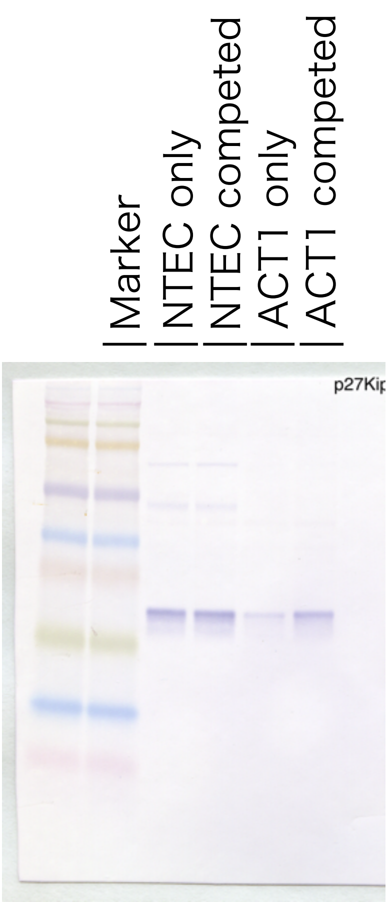

p27^{Kip1}

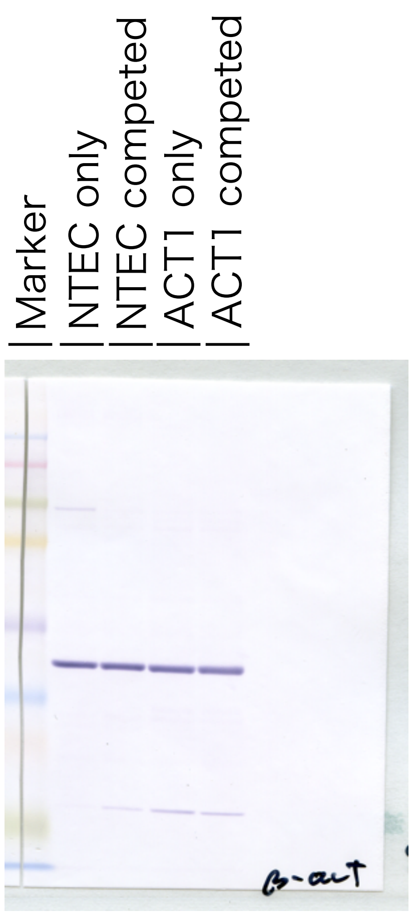

β-Actin

These images are for Figure 8

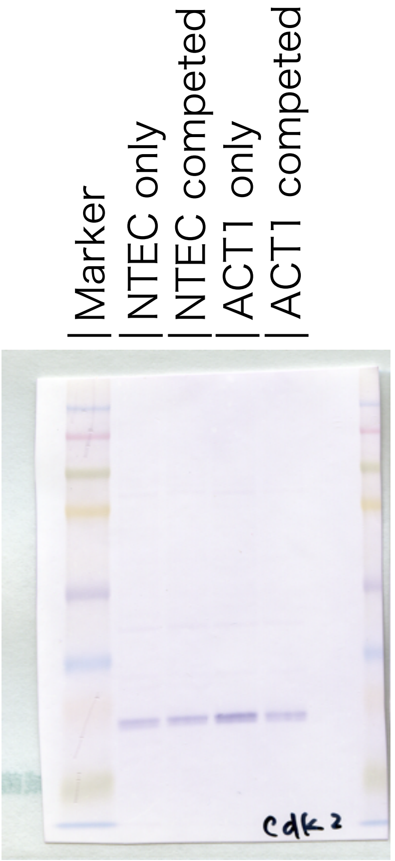

Cdk2

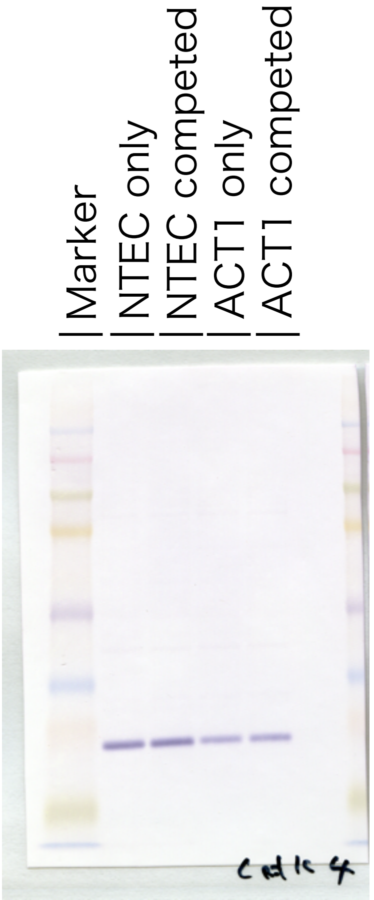

Cdk4

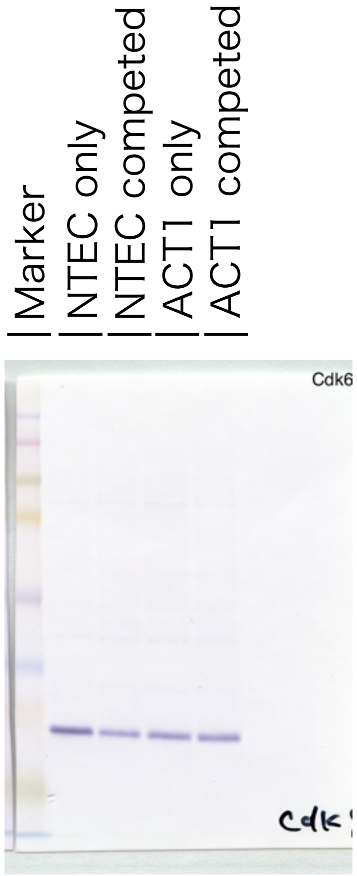

Cdk6

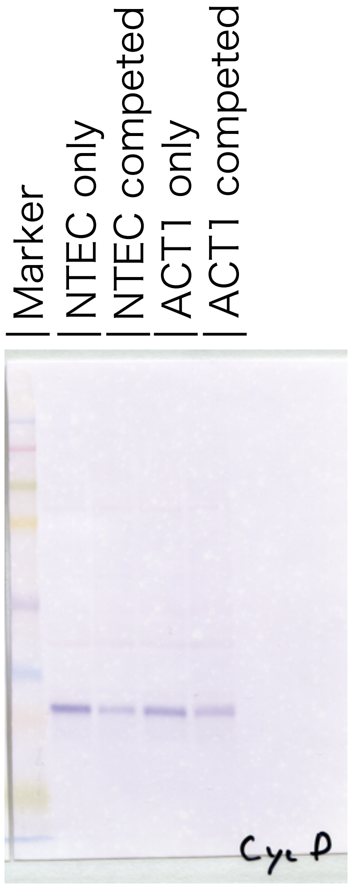

Cyclin D

These images are for Figure 8

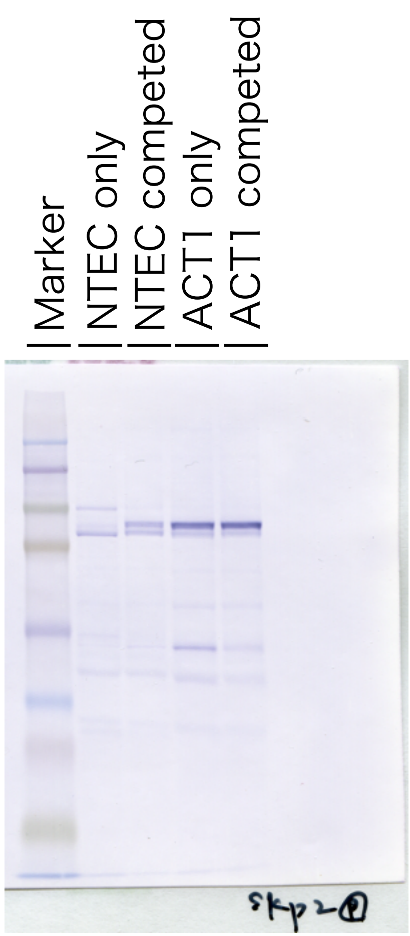

Skp2 S64 ⓟ

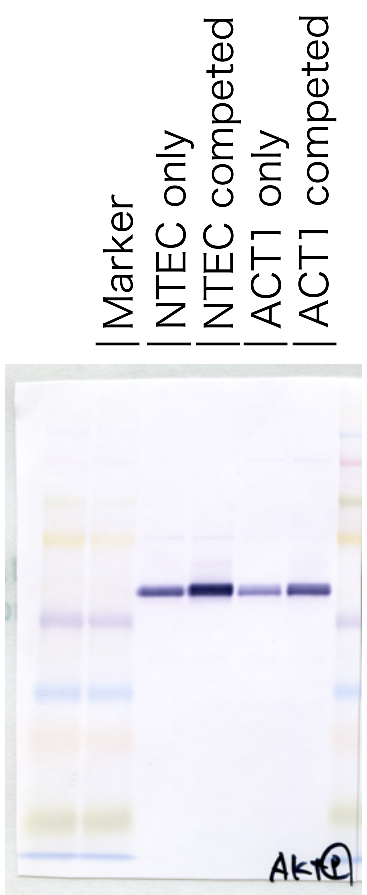

Akt S473/T308ⓟ

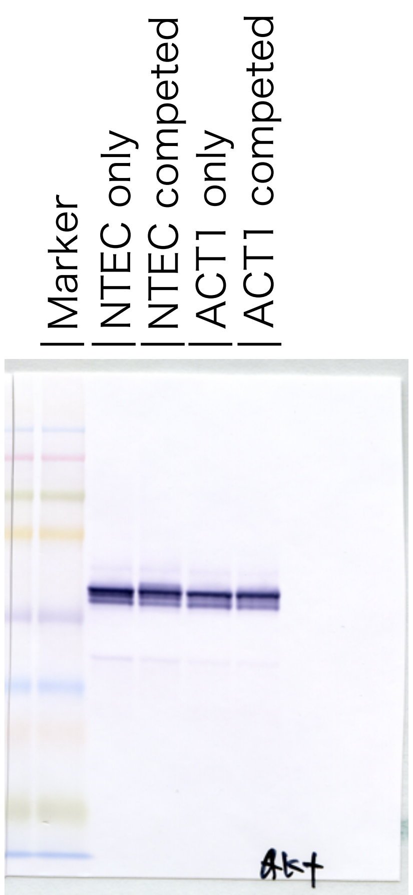

Akt

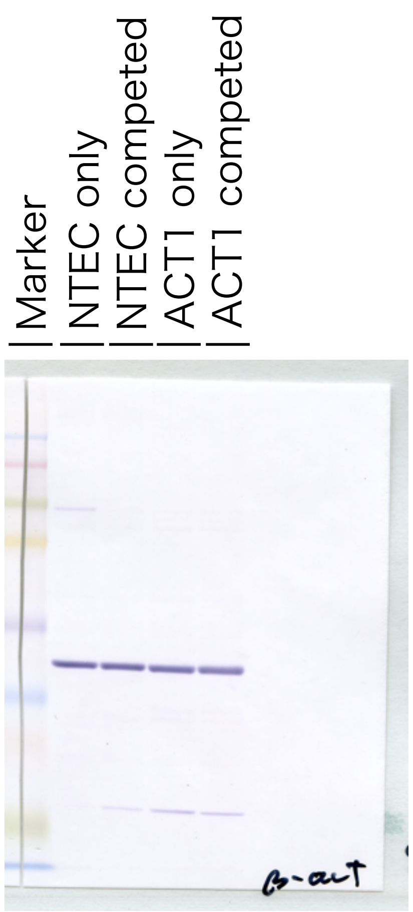

β-Actin

These images are for Figure 9

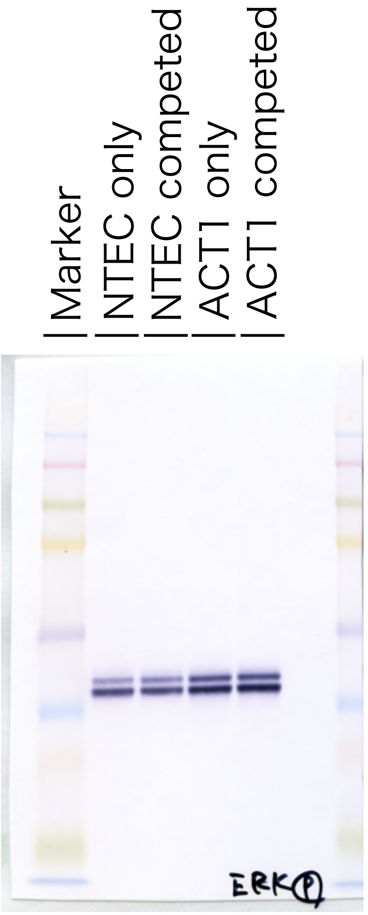

ERK1/2 ⓟ

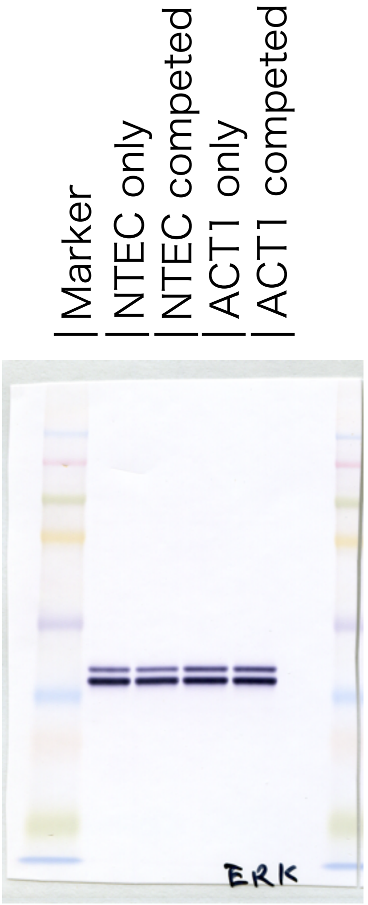

ERK1/2

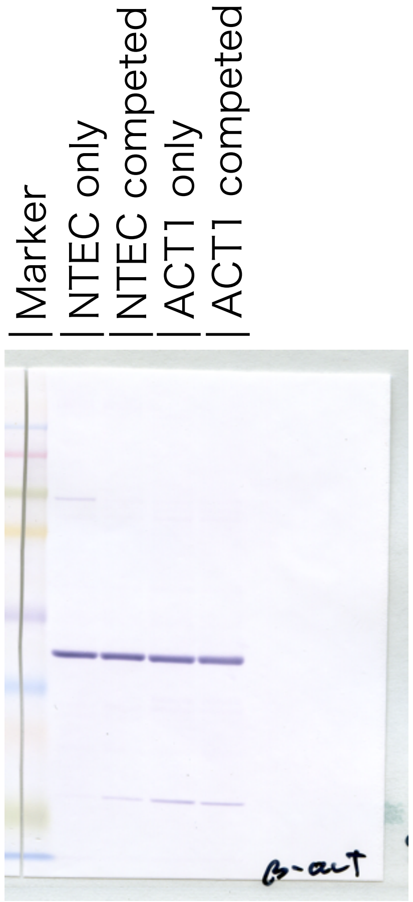

β-Actin

These images are for Figure 9

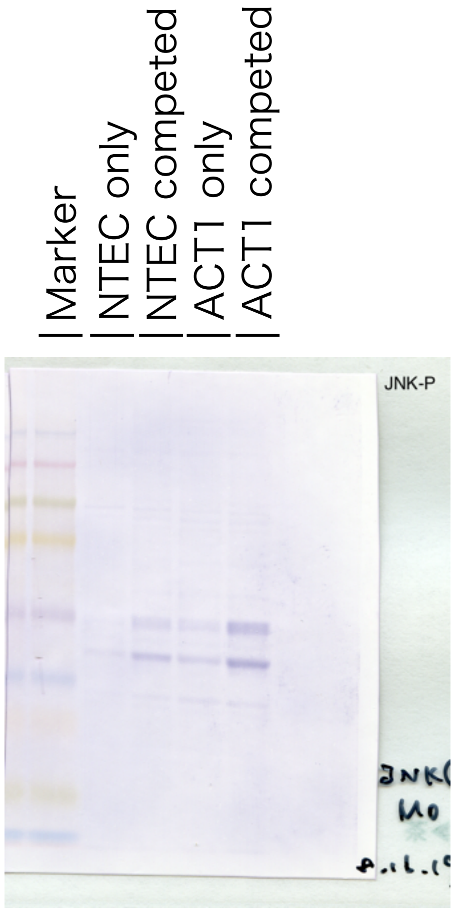

JNK1/2 ⓟ

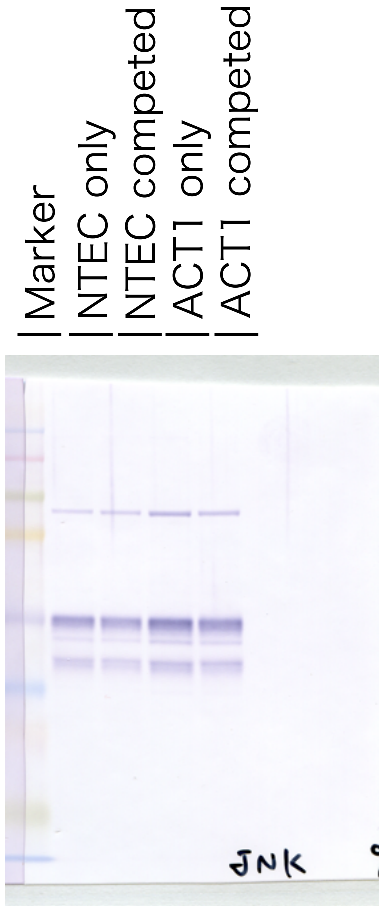

JNK1/2

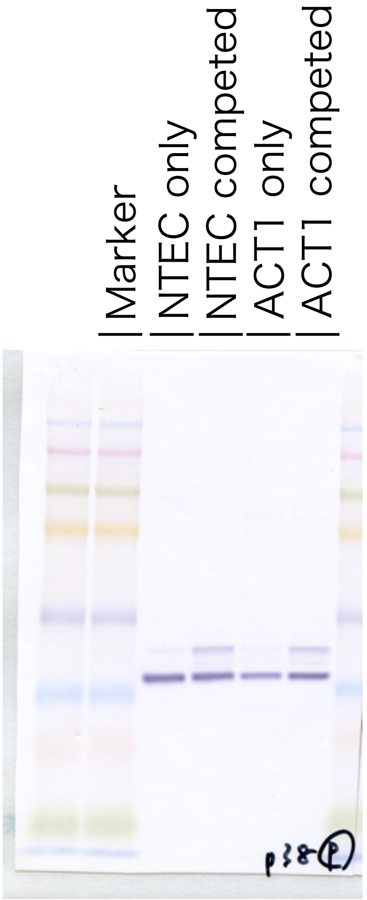

p38 ⓟ

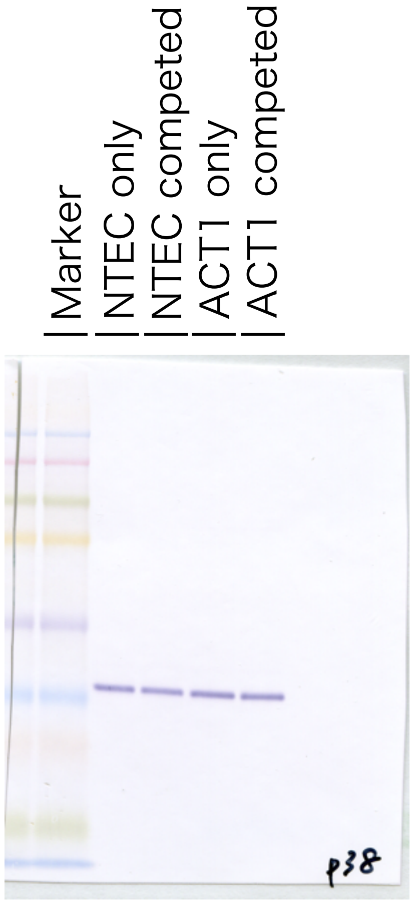

p38

These images are for S13 Fig

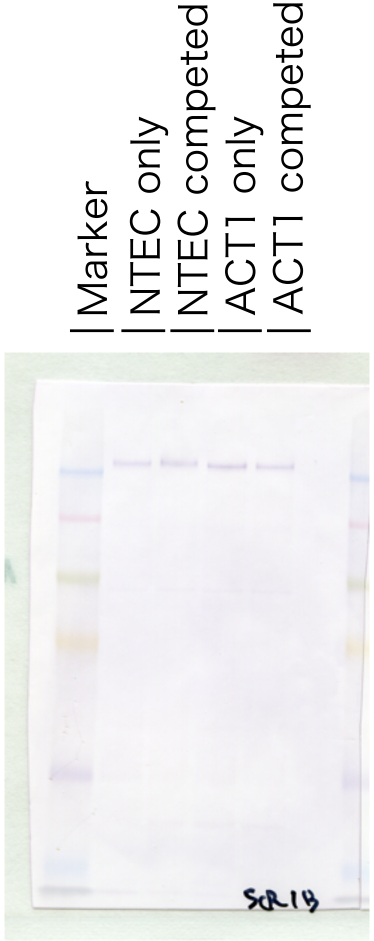

Scrib

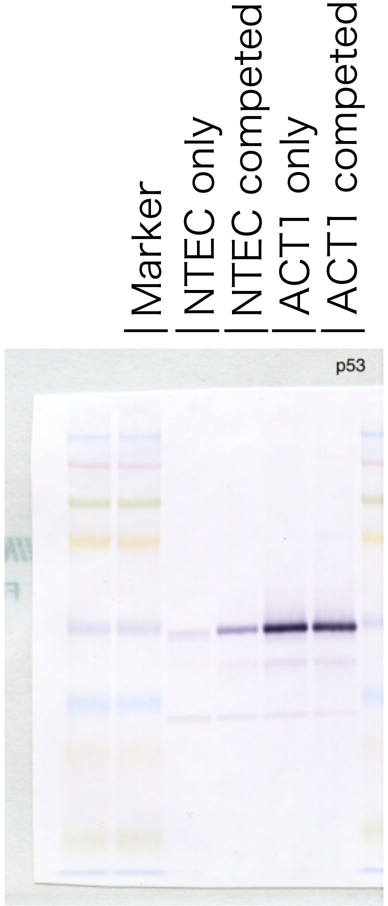

p53

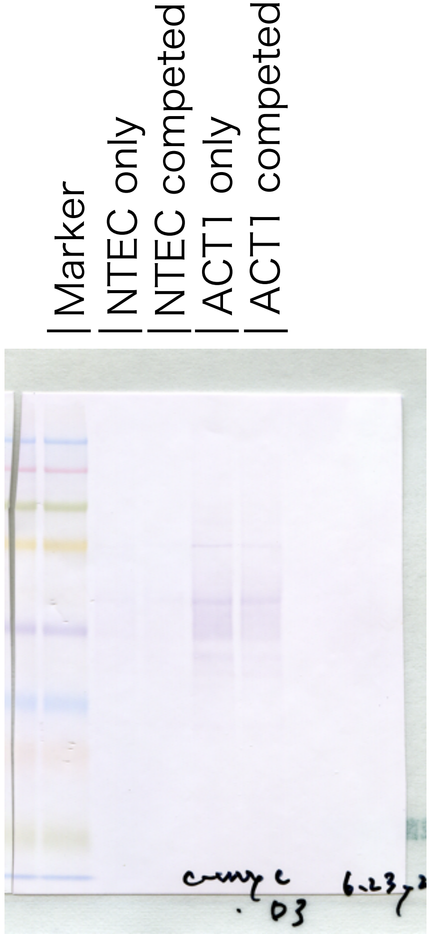

c-myc
